# Peptidomics-Driven Strategy Reveals Peptides and Predicted Proteases Associated With Oral Cancer Prognosis

**DOI:** 10.1074/mcp.RA120.002227

**Published:** 2020-12-03

**Authors:** Leandro Xavier Neves, Daniela C. Granato, Ariane Fidelis Busso-Lopes, Carolina M. Carnielli, Fábio M. de Sá Patroni, Tatiane De Rossi, Ana Karina Oliveira, Ana Carolina P. Ribeiro, Thais Bianca Brandão, André Nimtz Rodrigues, Pammela Araujo Lacerda, Miyuki Uno, Nilva K. Cervigne, Alan Roger Santos-Silva, Luiz Paulo Kowalski, Marcio Ajudarte Lopes, Adriana F. Paes Leme

**Affiliations:** 1 Brazilian Biosciences National Laboratory, National Center for Research in Energy and Materials, Campinas, Brazil; 2 Molecular Biology and Genetic Engineering Center, University of Campinas, Campinas, Brazil; 3 Dental Oncology Service, São Paulo Cancer Institute, São Paulo, Brazil; 4 Department of Head and Neck Surgery, Faculty of Medicine of Jundiaí, Jundiaí, Brazil; 5 Department of Internal Medicine, Molecular Biology and Cell Culture Laboratory, Faculty of Medicine of Jundiaí, Jundiaí, Brazil; 6 Center for Translational Research in Oncology, São Paulo Cancer Institute, São Paulo, Brazil; 7 Oral Diagnosis Department, Piracicaba Dental School, University of Campinas, Piracicaba, São Paulo, Brazil; 8 Head and Neck Surgery, Faculty of Medicine, University of São Paulo, São Paulo, Brazil

**Keywords:** saliva, head and neck squamous cell carcinoma, peptidomics, proteolysis, AHSG, alpha-2-HS-glycoprotein (fetuin A), C4BPA, C4b-binding protein alpha chain, CTS, cathepsin, FDR, false discovery rate, GO, Gene Ontology, HCl-SPE, hydrochloric acid saliva treatment followed by solid-phase extraction of peptides, ITIH2, inter-alpha-trypsin inhibitor heavy chain H2, LC-MS/MS, liquid chromatography–tandem mass spectrometry, LCN1, lipocalin-1, MeCN, acetonitrile, MUC7, mucin-7, N, clinically confirmed presence (N+) or absence (N0) of lymph node metastasis, NPEPPS, puromycin-sensitive amino peptidase, OSCC, oral squamous cell carcinoma, pN, pathologically confirmed presence (pN+) or absence (pN0) of lymph-node metastasis, PON1, paraoxonase/arylesterase-1, PRPs, proline-rich proteins, ROC-AUC, area under the receiver operating characteristic curve, SPINK5, serine protease inhibitor Kazal-type 5, SRM-MS, selected reaction monitoring–mass spectrometry, TCGA, The Cancer Genome Atlas, UF-SPE, ultrafiltration of saliva followed by solid-phase extraction of peptides

## Abstract

Protease activity has been associated with pathological processes that can lead to cancer development and progression. However, understanding the pathological unbalance in proteolysis is challenging because changes can occur simultaneously at protease, their inhibitor, and substrate levels. Here, we present a pipeline that combines peptidomics, proteomics, and peptidase predictions for studying proteolytic events in the saliva of 79 patients and their association with oral squamous cell carcinoma (OSCC) prognosis. Our findings revealed differences in the saliva peptidome of patients with (pN+) or without (pN0) lymph-node metastasis and delivered a panel of ten endogenous peptides correlated with poor prognostic factors plus five molecules able to classify pN0 and pN+ patients (area under the receiver operating characteristic curve > 0.85). In addition, endopeptidases and exopeptidases putatively implicated in the processing of differential peptides were investigated using cancer tissue gene expression data from public repositories, reinforcing their association with poorer survival rates and prognosis in oral cancer. The dynamics of the OSCC-related proteolysis were further explored via the proteomic profiling of saliva. This revealed that peptidase/endopeptidase inhibitors exhibited reduced levels in the saliva of pN+ patients, as confirmed by selected reaction monitoring–mass spectrometry, while minor changes were detected in the level of saliva proteases. Taken together, our results indicated that proteolytic activity is accentuated in the saliva of patients with OSCC and lymph-node metastasis and, at least in part, is modulated by reduced levels of salivary peptidase inhibitors. Therefore, this integrated pipeline provided better comprehension and discovery of molecular features with implications in the oral cancer metastasis prognosis.

## Highlights

• Proteolysis is accentuated in the saliva of patients with OSCC and lymph-node metastasis.
• Differential saliva peptidome outperformed proteomics in patient classification.
• Proteases implicated in differential proteolysis correlated with prognostic factors.
• Reduced levels of protease inhibitors disturb the proteolytic balance in the saliva.

## Graphical Abstract

**Figure:**
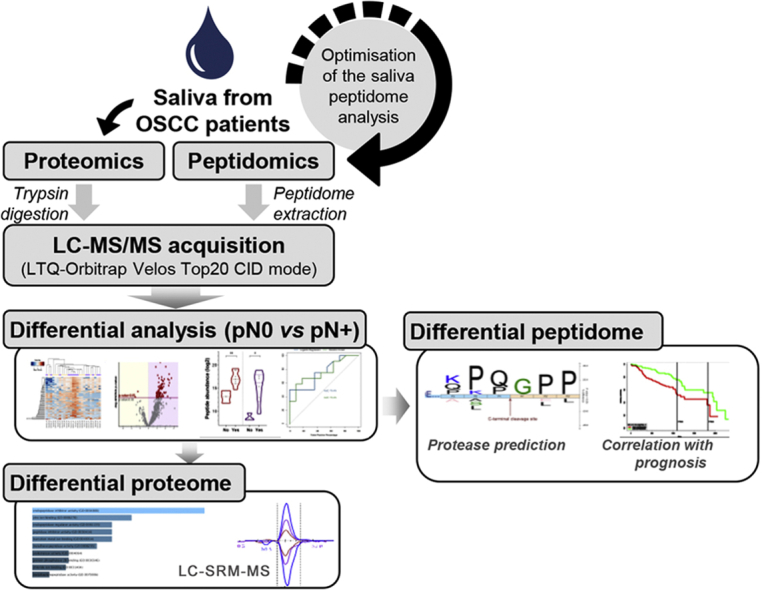

## In Brief

Mass spectrometry–based peptidomics has been applied to the saliva of patients with oral squamous cell carcinoma, enabling the discovery of signatures correlated with poor prognostic factors, including lymph-node metastasis. Sequence analysis of differential peptides allowed the prediction of proteases implicated in endogenous proteolysis and associated with prognosis in head and neck cancers. The results of combined saliva peptidomics and proteomics revealed that the accentuated proteolysis in patients with oral squamous cell carcinoma and lymph-node metastasis concurs with reduced levels of protease inhibitors in saliva.

Proteolysis is an irreversible protein modification involved in different cellular processes under physiological conditions (1). However, increased proteolytic activity is implicated in numerous diseases including cancer pathogenesis, as demonstrated by the prominent role of proteases in tumor growth, angiogenesis, invasion, and metastasis (2). Consequently, proteases have been investigated for diagnosis, prognosis, and therapeutic purposes in cancer (3, 4, 5).

Proteomic technologies have been shown to be useful to uncover molecular changes in protein or peptide abundances that may assist cancer diagnosis and prognosis by providing information that cannot be assessed by the routine clinical evaluation (6, 7). Noteworthy, peptidomic methods can complement the results from conventional bottom-up proteomics by providing insights on relevant proteolytic events (8) often lost during *in vitro* trypsin digestion. Therefore, the analysis of endogenous peptides from saliva may provide a new layer of information useful in the discovery of oral squamous cell carcinoma (OSCC) prognostic markers.

Peptidome analysis by liquid chromatography–tandem mass spectrometry (LC-MS/MS) can be challenging because of nonspecific protease digestion during sample preparation and computational processing of data, as well as difficulties in the biological interpretation (9). Among the methods that have emerged, the terminal amine isotopic labeling of substrates can be highlighted as a strategy that allows mapping *in vivo* processing of protein termini (10). Alternatively, the development of robust *in silico* analysis tools has also permitted the reconstruction of cleavage sites to predict active peptidases according to their known specificity or peptide libraries (11, 12). Thereby, the identification and analysis of endogenous peptides from complex biological samples such as liquid biopsies (*e.g.*, plasma/serum, urine, and saliva) has proved to be feasible (13, 14).

Therefore, considering the need for molecular markers to assist OSCC prognosis and the relevance of proteolytic process in cancer progression, we developed a simple and robust pipeline to analyze endogenous peptides and proteins from the saliva of patients with oral cancer. From mass spectrometry data, a sequential computational framework was established enabling cleavage site analysis, prediction of proteases, and correlation of their gene expression levels with OSCC prognosis using The Cancer Genome Atlas (TCGA) cancer database. Peptidome and proteome profiling uncovered subsets of molecules correlated with prognostic factors and was able to distinguish patients with and without lymph node metastasis. Thereby, our approach delivered a multifaceted view of proteolytic events taking place in the oral cavity of patients with OSCC with prognostic utility.

## Experimental Procedures

### Experimental Design and Statistical Rationale

A total of 79 saliva samples collected from patients with OSCC before any therapeutic intervention were used in this discovery study. Samples were divided into two groups of individuals diagnosed with (N+, *n* = 44) or without (N0, *n* = 35) cervical lymph node metastasis, a feature with major relevance in OSCC prognosis. Twenty-five samples were analyzed via peptidomics (cohort 1, pN0, *n* = 12; pN+, *n* = 13), 14 via bottom-up proteomics (cohort 2, pN0, *n* = 9 and pN+, *n* = 5), and 40 (cohort 3, N0, *n* = 14 and N+, *n* = 26) via Tier 2 exploratory targeted analysis (selected reaction monitoring–mass spectrometry [SRM-MS]), according to the availability of samples in our biorepository. All patients from cohorts 1 and 2 had pathological confirmation of lymph node metastasis, whereas classification of cohort 3 was predominantly clinical. In the discovery phases—that is, peptidomics, cohort 1, and bottom-up proteomics, cohort 2—samples were analyzed as single replicates, and a two-group comparison (pN0 *versus* pN+) was performed by Perseus, v1.6.10.45, using ANOVA (α = 0.05). Multiple comparison corrections were not applied at this stage because the consistency of observed differences was also evaluated through correlation analysis and receiver operating characteristic (ROC) curves (using R environment) or SRM-MS assay. Correlation analysis was performed to evaluate associations between the levels of endogenous peptides and proteins with anatomopathological data with prognostic utility. In addition, ROC curves were used to investigate proteins or peptides with acceptable power to discriminate pN0 and pN+ patients; area under the receiver operating characteristic curve (ROC-AUC) threshold was set to 0.7 in accordance with Hosmer Jr *et al.* (15). For SRM-based result verification, 40 samples (cohort 3) were analyzed as three technical replicates. The abundance of targets was normalized by internal standard spikes of stable labeled synthetic peptides and then compared between N0 and N+ groups using nested ANOVA (α = 0.05)—to account for technical and biological variations—and the Mann–Whitney test (α = 0.05)—for comparison of group averages. Patients' clinical information is available in supplemental Table S1. Run order was randomized using R (v3.4.0) environment to prevent systematic bias during MS acquisition (supplemental Table S2). Two patients with tumor recurrence and lymphoma were not considered in the final analysis of peptidomics data (*i.e.*, cohort 1; supplemental Table S3).

### Saliva Collection

Clinical samples were provided by Biobanco-Rede Premium, Faculdade de Medicina/Instituto de Câncer do Estado de São Paulo (cohorts 1 and 3), Faculdade de Medicina de Jundiai (cohort 2), and Faculdade de Odontologia de Piracicaba (cohorts 1 and 3). All procedures presented in this study were performed in accordance with the approved guidelines, patient informed consent, and institutional research ethics committees through Plataforma Brasil Certificado de Apresentação de Apreciação Ética #61402116.8.0000.0065 and #30658014.1.1001.0065 (Instituto de Câncer do Estado de São Paulo), #45091715.1.0000.5412 (Faculdade de Medicina de Jundiai), and #1.175.243 (Faculdade de Odontologia de Piracicaba). Saliva was collected during the morning period from individuals who had not eaten or ingested liquids (except for water) and had not performed oral hygiene for at least the past 1 h. Donor individuals were first instructed to perform a mouth wash with 5 ml of water, and saliva was collected without stimulation in a 15-ml falcon tube, according to a previously published protocol (16). Saliva aliquots were stored at −80 °C.

### Preparation of Saliva for Peptidomics

One milliliter of saliva was centrifuged at 500*g* for 10 min, 4 °C, to sediment cells and then the supernatant diluted to 30 ml in ice-cold PBS, pH 7.4, containing 1-mM PMSF and 5-mM EDTA. Ultracentrifugation at 110,000*g*, 4 °C, was performed for 1 h for recovery of supernatants comprising the saliva soluble fraction. Two methods for peptide extraction were evaluated using a test sample before processing the patients' saliva. In the first protocol (hydrochloric acid saliva treatment followed by solid-phase extraction of peptides [HCl-SPE]), samples were acidified with HCl to pH 2 (approximately 40-mM final concentration), after centrifugation at 12,800*g*, for 20 min, at 4 °C. The supernatant was recovered and peptides purified by solid-phase extraction (Sep-Pak C_18_, 500 mg; Waters), as described elsewhere (17). The eluted peptides were dried in SpeedVac (Thermo Scientific) and desalted using StageTips C_18_ (18). The second method was based on the enrichment of the peptidome by ultrafiltration (ultrafiltration of saliva followed by solid-phase extraction of peptides [UF-SPE]) upon a 3-kDa cutoff. First, urea and DTT were added to the samples at the final concentrations of 4 M and 10 mM, respectively. After centrifugation at 12,800*g*, for 20 min, at 4 °C, the clarified supernatant was transferred to Amicon Ultra-15 molecular weight cut-off 3-kDa tubes (Merck Millipore), to proceed with filtration at 4000*g*, at 4 °C, for approximately 90 min. The filtered solution enriched with the low-molecular-weight proteome was acidified with TFA to 0.5% final concentration (pH ∼2) and then submitted to solid-phase extraction by C_18_-reversed phase chromatography (Sep-Pak C_18_, 500 mg; Waters), as performed previously and then vacuum dried. Peptides were solubilized in 0.1% formic acid, and the final yield of the recovered peptides was assessed using the Quantitative Colorimetric Peptide Assay (Thermo Scientific). The method with best performance in terms of the number of peptide-spectrum match, unique sequences, and proteins identified was used to prepare the saliva samples obtained from patients with oral cancer. Extraction methods were compared under optimized LC-MS/MS configuration detailed in the following sections.

### Preparation of Saliva for Bottom-Up Proteomics and SRM-MS

Saliva was centrifuged for 5 min at 1500*g*, 4 °C, to pellet intact cells and debris. The protein concentration was determined on the supernatant using the Bradford assay kit (Bio-Rad). An aliquot with 10 μg of total protein was digested in the solution using trypsin, essentially as described elsewhere (19). In brief, samples were treated with urea buffer (100-mM Tris-HCl, pH 7.5, 8-M urea, 2-M thiourea, 5-mM EDTA, 1-mM PMSF, and 1-mM DTT) containing cOmplete Mini Protease Inhibitor Cocktail (Roche) and the mixture sonicated in an ultrasound bath for 10 min. After centrifugation at 10,000*g* for 5 min, the supernatants were sequentially treated with 5-mM DTT (for 25 min, at 56 °C) and 14-mM iodoacetamide (for 30 min in the dark, at RT) for protein reduction and alkylation of cysteines. The mixture was then diluted with 50-mM ammonium bicarbonate to lower urea concentration to 1.6 M and calcium chloride added to 1-mM final concentration. Samples were digested for 16 h at 37 °C using sequencing grade-modified trypsin (Promega) at a 1:50 enzyme-to-protein ratio. After overnight incubation, more trypsin (1:50) was added to samples prepared for bottom-up analysis, and digestion was continued for 5 to 6 h. The reaction was terminated by acidification to 0.4% TFA. Peptides were desalted with StageTips C_18_ (3 M Empore), dried in a vacuum concentrator, and reconstituted in 0.1% formic acid.

### LC-MS/MS Analysis for Peptidomics and Bottom-Up Proteomics

Tryptic and endogenous saliva peptides were analyzed using the EASY-nLC II (Proxeon Biosystems) coupled to the LTQ-Orbitrap Velos mass spectrometer (Thermo Fisher Scientific). Approximately 750 ng or 2 μg of peptides, for peptidomics or proteomics, respectively, were resolved by reversed-phase chromatography using the analytical column PicoFrit C_18_ (20 cm × 75 μm id, 5 μm; New Objective) at a constant flow rate of 300 nl/min. For peptidomics, a round of gradient optimization was performed using the following ramps of phases A (0.1% formic acid in water) and B (0.1% formic acid in acetonitrile [MeCN]): method 1 (M1), 10 to 30% B over 27 min and then 30 to 45% B in 5 min; method 2 (M2) 2 to 20% B over 25 min and then 20 to 30% B in 7 min; method 3 (M3) 2 to 30% B over 27 min and then 30 to 45% in 5 min. All ramp configurations were followed by identical washing and conditioning steps. For proteomics, tryptic digests were resolved over a 212-min gradient (2–90% B; 35% B at 175 min). Eluting peptides were analyzed by the mass spectrometer operating in a positive mode. Data-dependent acquisition of saliva peptidome and tryptic digest was performed using similar parameters, except for the minimum charge state of peptides selected for MS/MS analysis, +1 or +2, for endogenous or tryptic peptides, respectively. In both approaches, precursor ions (*m*/*z* 300–1600) were scanned in the Orbitrap with resolution defined to r = 60,000 and 1E6 target ions. Up to 20 most intense ions (5E4 target ions) were isolated through a 3-Da window and activated by collision-induced dissociation, with normalized collision energy of 35%, activation Q = 0.25, and activation time of 10 ms. Product ions were detected by the ion trap operating in the normal scan rate. Dynamic exclusion was enabled with an exclusion list of up to 500 ions, an exclusion duration of 60 s, and a count repetition of 1.

### Processing of Peptidomics Data

*De novo* sequencing–assisted database searching (20) was performed using PEAKS Studio X (Bioinformatics Solutions Inc). Search parameters included unspecific proteolysis, enabling peptides with up to 65 residues to be matched, and mass error tolerance of 10 ppm and 1 Da for precursor and product ions, respectively. Oxidation of methionine residues (+15.99 Da) and acetylation of proteins N-termini (+42.01 Da) were allowed as variable modifications. Peptide sequences matching the Human UniProt database (93,599 protein sequences, 36,574,184 residues, release June 2018) were obtained at a false discovery rate (FDR) ≤ 0.01 for both proteins and peptides. FDR estimates were calculated using the “decoy-fusion” method built in the PEAKS Studio software (20). Quantitative data were generated by the PEAKS Q module using normalized precursor intensity extracted using a 10-ppm tolerance and a 2-min retention time window. PEAKS Q quality threshold was adjusted to 8 to remove lower-quality quantitative features, and only peptides with at least one valid value in both pN0 and pN+ groups were considered for relative quantification.

### Processing Bottom-Up Proteomics Data

Raw data were processed using MaxQuant v1.5.8.0 software (https://www.maxquant.org/), and MS/MS spectra were searched against The Human UniProt database (93,599 protein sequences, 36,574,184 residues, release June 2018) using the Andromeda search engine. Search parameters included tolerance of 6 ppm for precursor ions and 0.5-Da for product ions, trypsin/P enzyme specificity with a maximum of two missed cleavages. Carbamidomethylation of cysteine (+57.02) was considered a fixed modification, and oxidation of methionine (+15.99) and protein N-terminal acetylation (+42.01) were considered variable modifications. The FDR was estimated using the target-decoy method (21) and set to ≤0.01 for both the protein and peptide matches. Protein quantification was performed using the LFQ algorithm implemented in MaxQuant software, with a minimal ratio count of one and a 2-min window for matching between runs. Identified protein entries were processed excluding reverse sequences and those identified “only by site.”

### Association of Peptide and Protein Abundances With Clinical Data

Linear regression analysis was performed using the R (v3.6.0) environment to evaluate the relationship between endogenous peptide and protein abundances with clinicopathological features, namely, the size of tumor, pathologically confirmed presence and stage of lymph nodal metastasis (pN), differentiation, extracapsular extension, worse pattern of invasion, perineural invasion, and blood/lymphatic vessel invasion. Linear regression with *p*-value ≤ 0.05 was used to define significance. The Pearson product–moment correlation coefficient (denoted by *R*) was also calculated to measure the strength of the association between protein and peptide abundances and clinicopathological features. Peptides with *R* < −0.7 or 0.7 < *R*, *R*^2^ > 0.5, at least 6 valid values per group, and ≥3 valid values per clinical feature were considered.

### ROC Curve by Logistic Regression and Random Forest Analysis

The power of each endogenous peptides and proteins as classifiers to discriminate pN0 and pN+ patients was evaluated by the construction of ROC curves using random forest (22) and logistic regression. Binary regression was used when multiple peptides were combined. The sensitivity and specificity were calculated using the peptide or protein intensities from label-free quantification experiments. The area under the curve (AUC-ROC) with a 95% confidence interval was used for comparison. Optimal cut-off by highest sensitivity (true-positive rate) in function of the specificity (false-positive rate) was calculated, and the decision threshold was assigned to the value of 70%. For all statistical comparisons, an ANOVA *p*-value ≤ 0.05 was used to define significance. The data analysis was performed using the package pROC (23) and the R environment, version 3.4.4.

### Cleavage Site Analysis of Endogenous Peptides and Protease Prediction

Differentially abundant peptides had their N- and C-terminal flanking residues mapped on full-length protein sequences (UniProt Human proteome, release June 2018) using DBToolKit, v4.2.4 (24) to reconstruct the cleavage sites. Amino acid frequency (P3-P3ʹ) was determined by IceLogo v1.0.2 (https://iomics.ugent.be/icelogoserver/) compared with natural occurrence in the human proteins deposited at SwissProt database. Over-representation of amino acids was considered when *p*-value was ≤0.05.

Protease prediction was carried out by Proteasix (http://proteasix.org/) tool (11) from the same subset of differential saliva peptides using the “observed” mode, that is, matching against cleavage site associations collected from the literature.

### Biological Characterization and Search for Hub Molecules

Proteases predicted to cleave the differential peptides (pN0 *versus* pN+) were submitted to over-representation test of Gene Ontology (GO) annotation of biological processes, molecular functions, and cellular component using DAVID (25). An interaction network (*p*-value < 0.05) was created using Contextual Hub Analysis Tool against Reactome database in Cytoscape 3.4.0 (26).

### Association of Predicted Proteases With Prognosis Using Database Repositories

The association of transcript levels of predicted proteases in the tissues of patients with a prognosis of oral cancer was evaluated using PROGgene v2 tool (27). This analysis was performed with the list of proteases predicted to cleave peptides differentially abundant between pN0 and pN+ patients. PROGgene generated survival curves considering the gene expression values from distinct databases available for head and neck tumors. More specifically, four databases were used to conduct the analysis, namely, TCGA (2015), GSE65858 (28), GSE27020 (29), and E-MTAB-1328 (30), providing clinical and molecular information from 291, 269, 108, and 89 patients with head and neck cancer, respectively. Median gene expression was used as a cut-off to determine low and high expression of selected markers, and significance threshold was set to *p*-value ≤ 0.05.

A survival analysis was also performed using Kaplan–Meier curves and the log-rank test. In the univariate survival analysis, the comparison was performed between the higher and the lower expression values. These were defined by group formation considering the log2 expression of the protease of interest in patients assessed from the TCGA database. Unbiased group assignment was achieved using *mclust* package (31) under R environment. For the multivariate survival analysis, the Cox proportional hazard model with a stepwise method was used. A *p*-value < 0.05 was set as the significance threshold in the Cox proportional hazard model (32). Data were tested for normality and homogeneity of variance using the Shapiro–Wilk test (*p*-value ≤ 0.05) to drive decisions of parametric or nonparametric tests for group comparison with the clinical categories.

### Development and Analytical Validation Targeted MS Assay

Proteotypic peptides of alpha-2-HS-glycoprotein (AHSG; fetuin A) and serine protease inhibitor Kazal-type 5 (SPINK5) were selected according to Gallien *et al.* and Lange *et al.* (33, 34). In brief, sequences unique to AHSG and SPINK5 protein products, fully tryptic (P1 = K/R), and <20 amino acids residues were selected from the peptide list provided by shotgun proteomics. Based on these criteria, two peptides (FSVVYAK and HTLNQIDEVK) were selected for fetuin A while seven candidates remained available for SPINK5. In that case, the list of SPINK5 peptide candidates was narrowed to a subset of three sequences (FFQSLDGIMFINK, ATAPTELNCDDFK, and EAVQELCSEYR) that also presented empirical evidence in our previous shotgun analysis OSCC saliva (16) and SRMatlas repository (http://www.srmatlas.org).

Relative quantification of fetuin A and SPINK5 across 40 OSCC saliva samples (cohort 3) was carried out in the presence of 0.8-5 pmol/ul surrogate stable isotope labeled peptides (Thermo Scientific) synthesized with a C-terminal [^13^C_6_,^15^N_2_]-lysine or [^13^C_6_,^15^N_4_]-arginine. In addition to fetuin A and SPINK5 surrogate peptides, samples were also spiked with the Pierce Retention Time Calibration Mixture (120 fmol on column; Thermo Scientific) for monitoring instrument stability during SRM-MS acquisition. Two SPINK5 surrogate peptides (ATAPTELNCDDFK and EAVQELCSEYR) were not detected during SRM assay development, thus were not included in the final analysis of cohort 3. The other target analytes were monitored using three transitions in the light and heavy channels (AHSG protein: FSVVYAK, *m*/*z* 407.22, +2, and [y3] *m*/*z* 381.21+, [y4] *m*/*z* 480.28+, [y5] *m*/*z* 579.35+; HTLNQIDEVK, *m*/*z* 598.82, +2, and [y2] *m*/*z* 246.18+, [y4] *m*/*z* 490.25+, [y5] *m*/*z* 603.33+; and SPINK5 protein: FFQSLDGIMFINK, *m*/*z* 780.40, +2 and [y2] *m*/*z* 261.15+, [y4] *m*/*z* 521.31+, [y5] *m*/*z* 652.35+). Transition selection was based on the ranking order provided by Skyline using data-dependent acquisition spectra.

Samples were analyzed on a Xevo TQ-XS triple quadrupole mass spectrometer (Waters), as described by Carnielli *et al.* (18). One microgram of saliva digest was resolved over a 60-min gradient using an Acquity UPLC-Class M equipped with a trap column (Waters Acquity UPLC BEH C18 130A, 5 μm, 300 μm × 50 mm) and a BEH Shield C18 IonKey column (10-cm × 150-μm ID packed with 1.7-μm C18 particles, Waters) at 1.2 μl/min flow rate and temperature set to 40 °C. MeCN gradient started at 2% B (MeCN, 0.1% formic acid), following a linear ramp to 40% B over 45 min, followed by a step increase to 85% B until 47 min and conditioning at 2% B until 60 min. Mass spectrometry analysis of eluting peptides was performed via SRM-MS, with quadrupoles Q1 and Q3 operating as unit mass resolution (0.7 Th full width at half maximum). Schedule SRM acquisition was adjusted to a 3-min elution window, with dwell times automatically set in MassLynx v4.2 to achieve at least ten points per peak over a 15-s elution profile. The optimal collision energy was determined for each peptide by Skyline. To avoid systematic bias in data acquisition, samples were randomized using the software R v3.4.0 (35) and analyzed in triplicate. A sample order in each replicate batch was randomized independently. The data analysis was manually performed in Skyline v20.1.0.76.

## Results

### Development of an Analytical Pipeline for the Study of the Salivary Peptidome

**Fig. 1:**
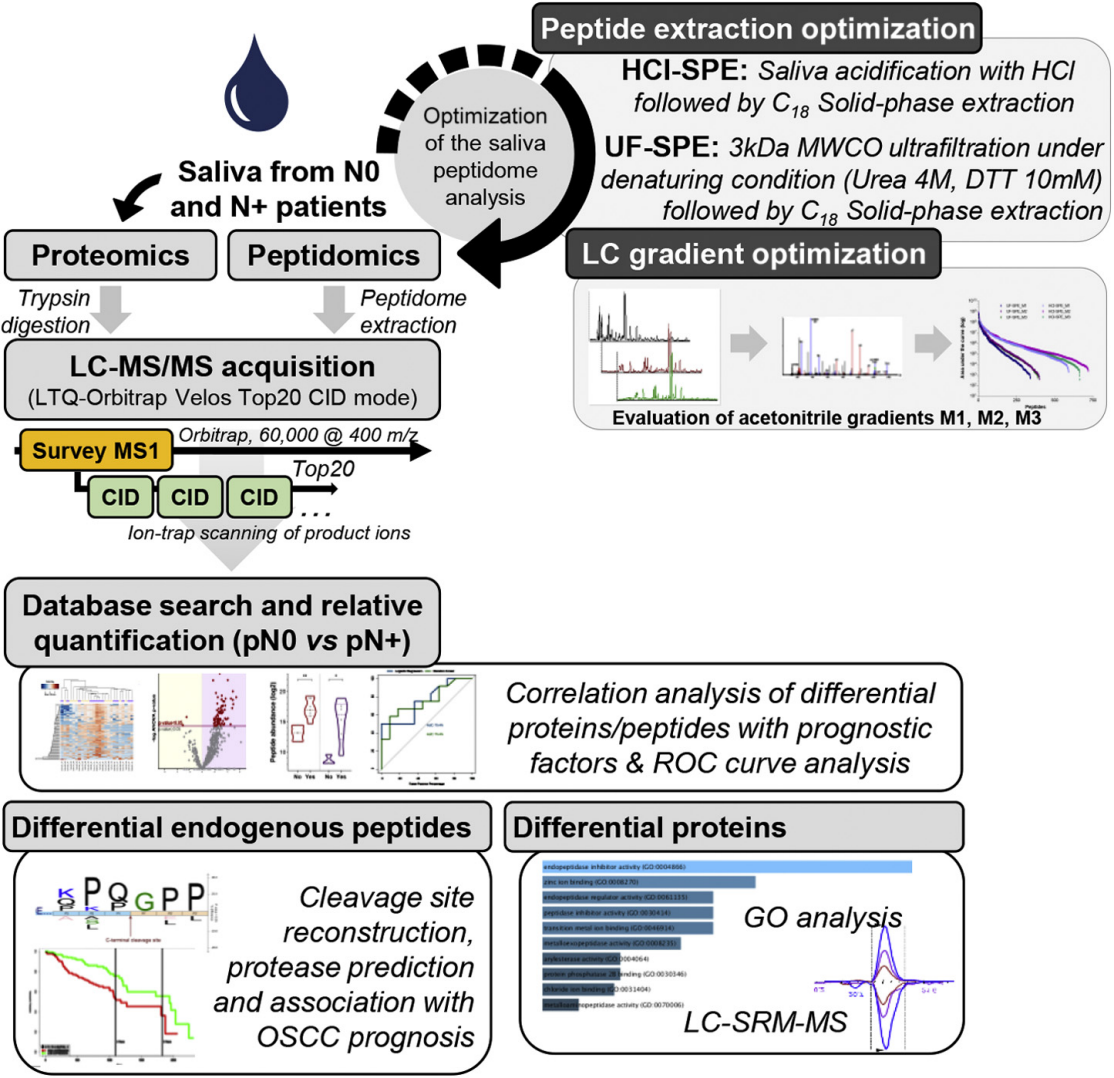
**Pipeline for characterization of saliva peptidome and proteome**. A pool of saliva (n = 3) was first used to optimize the methodology for peptide recovery and analysis via LC-MS/MS. Briefly, peptide fractions were recovered by (i) saliva acidification with HCl (pH 2) followed by C_18_ solid-phase extraction (HCl-SPE) or (ii) 3-kDa ultrafiltration under denaturing conditions (4 M urea, 10-mM DTT) followed by C_18_ solid-phase extraction (UF-SPE) of the flow through. LC-MS/MS analysis of HCl-SPE and UF-SPE was performed by testing three variations of a 45-min acetonitrile gradient (M1, M2, and M3) for improved peptide separation. Saliva from patients with oral squamous carcinoma (OSCC) and with and without lymph-node metastasis pathologically confirmed, pN0 and pN+, respectively, were prepared using the HCl-SPE protocol. Tandem spectrum data acquired using an LTQ-Orbitrap Velos operating in Top20 DDA mode using CID activation were processed in PEAKS Studio X—using an unspecific digest mode—or MaxQuant, for peptidomic and proteomics analyses, respectively. Differential molecules between pN0 *versus* 'pN+ group comparison were submitted to correlation tests with prognostic factors, such as invasiveness, differentiation, and extracapsular extension. Differential protein and peptides were also tested as classifiers of pN0 and pN+ patients using ROC curves. Cleavage site analysis revealed potential proteases implicated in the breakdown of saliva proteins and correlated with prognosis. Complementary proteomics analysis uncovered regulatory mechanisms of saliva proteolysis via peptidase, enzymes, and inhibitors levels, finally verified via SRM-MS. DDA, data-dependent acquisition; GO, Gene Ontology; HCl-SPE, hydrochloric acid saliva treatment followed by solid-phase extraction of peptides; LC-MS/MS, liquid chromatography–tandem mass spectrometry; ROC, receiver operating characteristic; SRM-MS, selected reaction monitoring–mass spectrometry; UF-SPE, ultrafiltration of saliva followed by solid-phase extraction of peptides.

Before the analysis of samples from patients with OSCC, we evaluated the performance of two extraction methods (HCl-SPE and UF-SPE) and three liquid chromatographic conditions (M1, M2, M3) to achieve improved coverage of the saliva peptidome (Fig. 1). The extraction methods explored physical-chemical features such as isoelectric precipitation, hydrophobicity, and molecular mass to separate endogenous peptides from intact soluble proteins. The first protocol (HCl-SPE) consisted of the direct loading of diluted and acidified saliva samples (pH ∼2) onto solid-phase C_18_ cartridges for peptide binding, whereas the second approach (UF-SPE) was based on centrifugal ultrafiltration followed by solid-phase extraction. Test samples were then evaluated using three LC-MS methods, varying on MeCN ramp configuration, to provide improved chromatographic conditions for peptide identification.

**Fig. 2:**
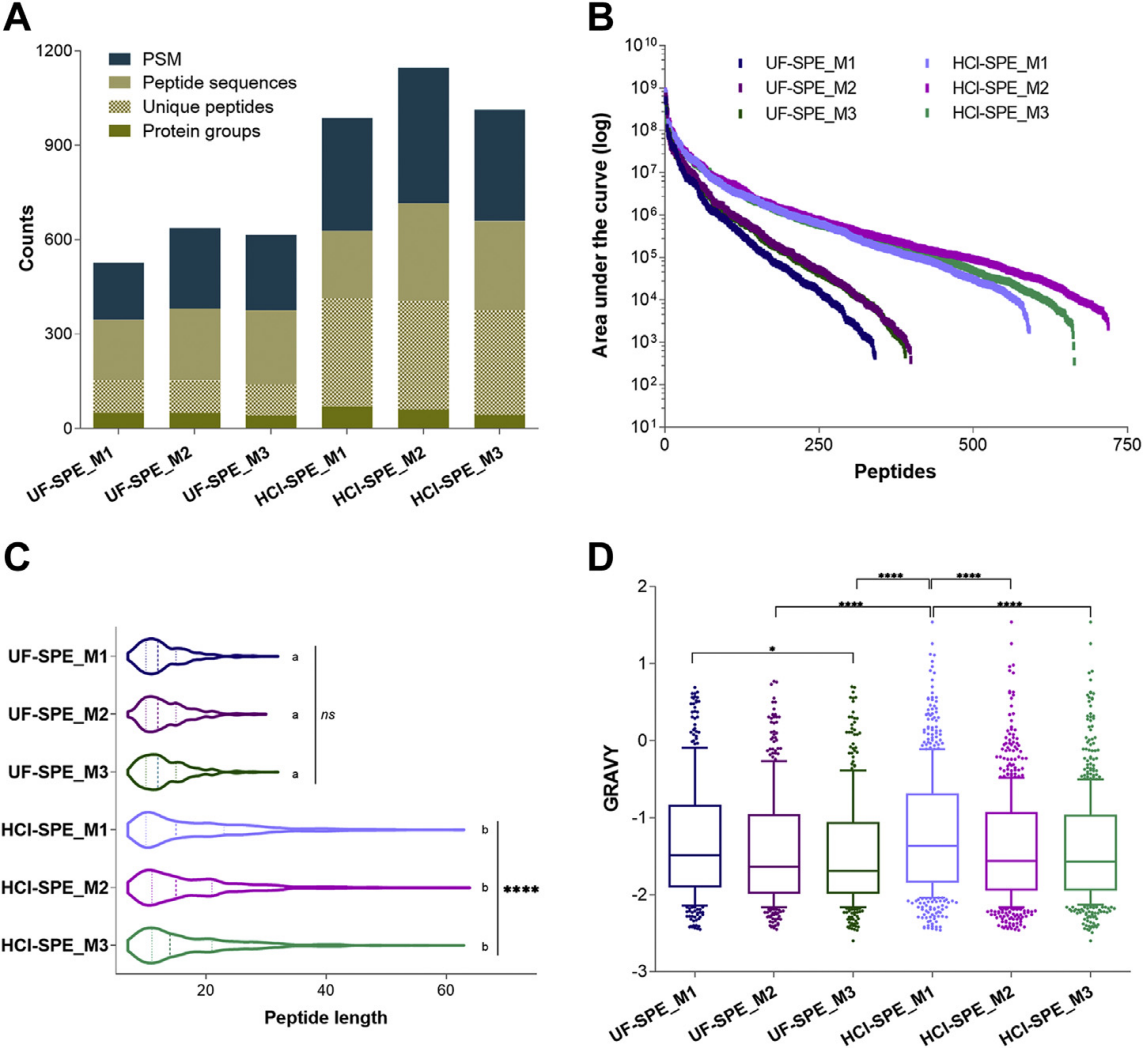
**Optimization of the saliva peptidome extraction and LC-MS/MS analysis**. Endogenous peptides recovered from saliva using either ultrafiltration (3-kDa MWCO; UF-SPE) or HCl acidification following C_18_ solid-phase extraction (HCl-SPE) were analyzed using three LC gradients (M1, M2, and M3) to achieve optimal chromatographic separation. *A*, samples prepared with the HCl-SPE extraction method had higher identification rates, particularly when the gradient M2 was used for resolving the peptidome. *B*, abundant range of peptides detected spanned over 5 to 6 orders of magnitude. *C*, the ultrafiltration-based method biased the analysis toward endogenous peptides <3 kDa while HCl-SPE extraction allowed the recovery of larger peptides. *D*, GRAVY values of peptides identified across the experimental conditions suggested that LC gradients played a major role in the identification of more hydrophobic peptides regardless of the extraction method used. Kruskal–Wallis with Dunn's tests indicated significant differences; ∗*p*-value < 0.05, ∗∗∗∗*p*-value < 0.0001. Box plot boundaries at 10th and 90th percentiles. GRAVY, grand average of hydropathy; HCl-SPE, hydrochloric acid saliva treatment followed by solid-phase extraction of peptides; LC-MS/MS, liquid chromatography–tandem mass spectrometry; MWCO, molecular weight cut-off; UF-SPE, ultrafiltration of saliva followed by solid-phase extraction of peptides.

Mass spectrometry analysis of the saliva peptidome resulted in the identification of peptides with ion intensities spanning over six orders of magnitude, regardless of the extraction methods or LC-MS gradient (Fig. 2, *A*–*B*). Under similar sample loads, the average number of peptides identified using HCl-SPE extraction (667 sequences) was 1.8× higher than the average observed in UF-SPE (367 sequences, Fig. 2, *A*–*B*; supplemental Tables S4 and S5). The HCl-SPE procedure also delivered more peptide-spectrum match (1.8× increase, 593 *versus* 1049) and unique peptides (2.7× increase, 149 *versus* 398; Fig. 2*B*). Notably, the increase in the number of proteins identified using HCl-SPE protocols was less pronounced (1.2×) compared with the other metrics, but the superior average ratio of unique peptides per protein exhibited in comparison to UF-SPE (7 *versus* 3) indicated a higher coverage of cleavage products per protein.

Peptide length analysis indicated that ultrafiltration clearly limited the size of peptides recovered, biasing the analysis to a subset of polypeptides containing no more than 27 to 30 amino acid residues (Fig. 2*C*). The distribution of grand average of hydropathy values across the methods shows that the extraction method used had minor influence on the hydrophobicity of peptides identified, unlike the chromatographic settings tested (Fig. 2*D*). Overall, the combination of HCl-SPE sample preparation strategy and the LC condition “M2” outperformed the other methodologies so that HCl-SPE_M2 was the method of choice for clinical samples analysis.

### Patients With OSCC and Nodal Metastasis Exhibit a Distinctive Salivary Peptidome

The successful development of a protocol suitable for saliva peptidome extraction and MS analysis allowed us to investigate peptides potentially associated with oral cancer prognostic factors such as lymph-node metastasis. For this purpose, the saliva peptidome from 25 patients with OSCC and with (pN+, n = 13) or without (pN0, n = 12) lymph-node metastasis was first characterized using mass spectrometry and then following a pipeline of bioinformatic analyses (Fig. 1). This included the prediction of proteases potentially associated with the generation of differential peptides between pN0 and pN+ saliva, and correlation analysis between prognostic features and peptide abundance. Moreover, the association between transcript levels of predicted proteases in tumor tissues and prognosis of patients with head and neck cancer was evaluated using PROGgene and data available in the TCGA repository.

**Fig. 3:**
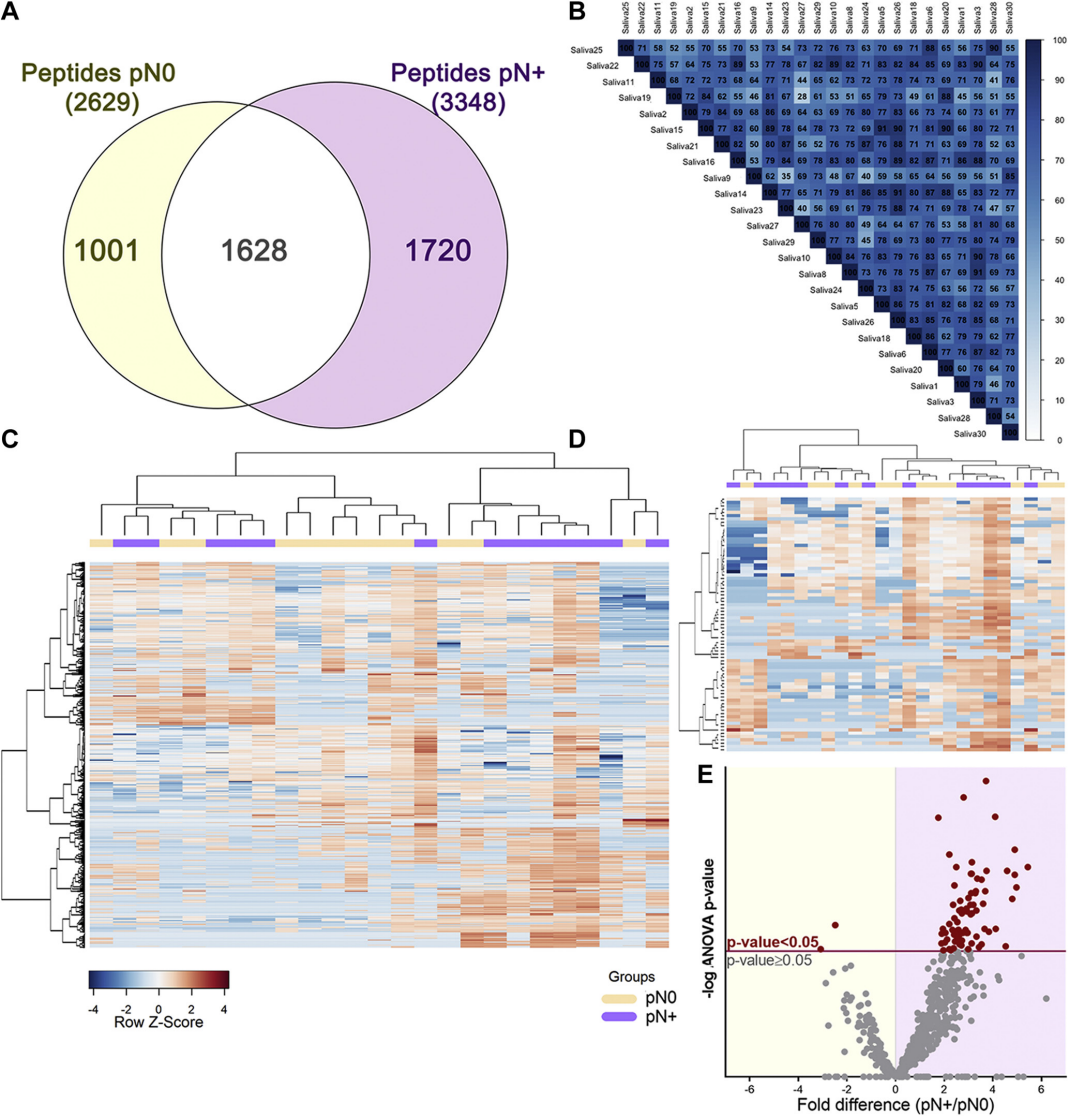
**Composition analysis of the saliva peptidome from pN0 and pN+ OSCC patients.***A*, qualitative analysis reveals a significant number of group-specific peptide matches and <40% overlap between pN0 and pN+ patients. *B*, the correlogram of the 25 LC-MS runs saliva peptidome showing Pearson's R ranging from 0.5 to 0.9. *C*, the heat map of the whole saliva peptidomes and (*D*) differentially abundant peptides (ANOVA *p*-value ≤ 0.05) show no clear grouping of samples based on a single classifier feature such as lymph node metastasis (N+/N0). Hierarchical clustering was performed using the Pearson's correlation and Ward's method. *E*, differential distribution of peptides quantified in pN0 and pN+ samples represented on a volcano plot. Peptides above the significance threshold (ANOVA *p*-value ≤ 0.05) are highlighted as *red dots*.

Of 4349 peptides identified in our data set (FDR ≤ 0.01; supplemental Table S6), 1720 and 1001 peptides were exclusive to pN+ and pN0 groups, respectively, while 1628 peptides were shared between the groups (Fig. 3*A*). Pearson correlation coefficients of the 25 LC-MS/MS runs ranged from 0.5 to 0.9, anticipating a dynamic salivary peptidome (Fig. 3*B*). After filtering lower-quality features in PEAKS Q (quality threshold = 8 and at least one valid value in both groups), label-free quantitative data of 676 peptides—mapping to 48 protein groups—were used to assess differences between pN0 and pN+ saliva (Fig. 3*C*). A subset of 77 peptides, assigned to 22 protein groups, was revealed to be differentially abundant (ANOVA *p*-value ≤ 0.05) in the saliva of patients diagnosed with lymph node metastasis (Fig. 3, *D*–*E*, supplemental Table S7). Despite group differences in peptide abundance, hierarchical clustered heat maps of both the whole saliva peptidome and differential peptides did not exhibit a clear separation toward pN+ and pN0 status. Therefore, we evaluated whether other prognostic features could be also associated with the differences observed in the saliva peptidome.

### Peptide Abundance in Saliva Correlates With Clinicopathological Prognostic Factors

**Fig. 4:**
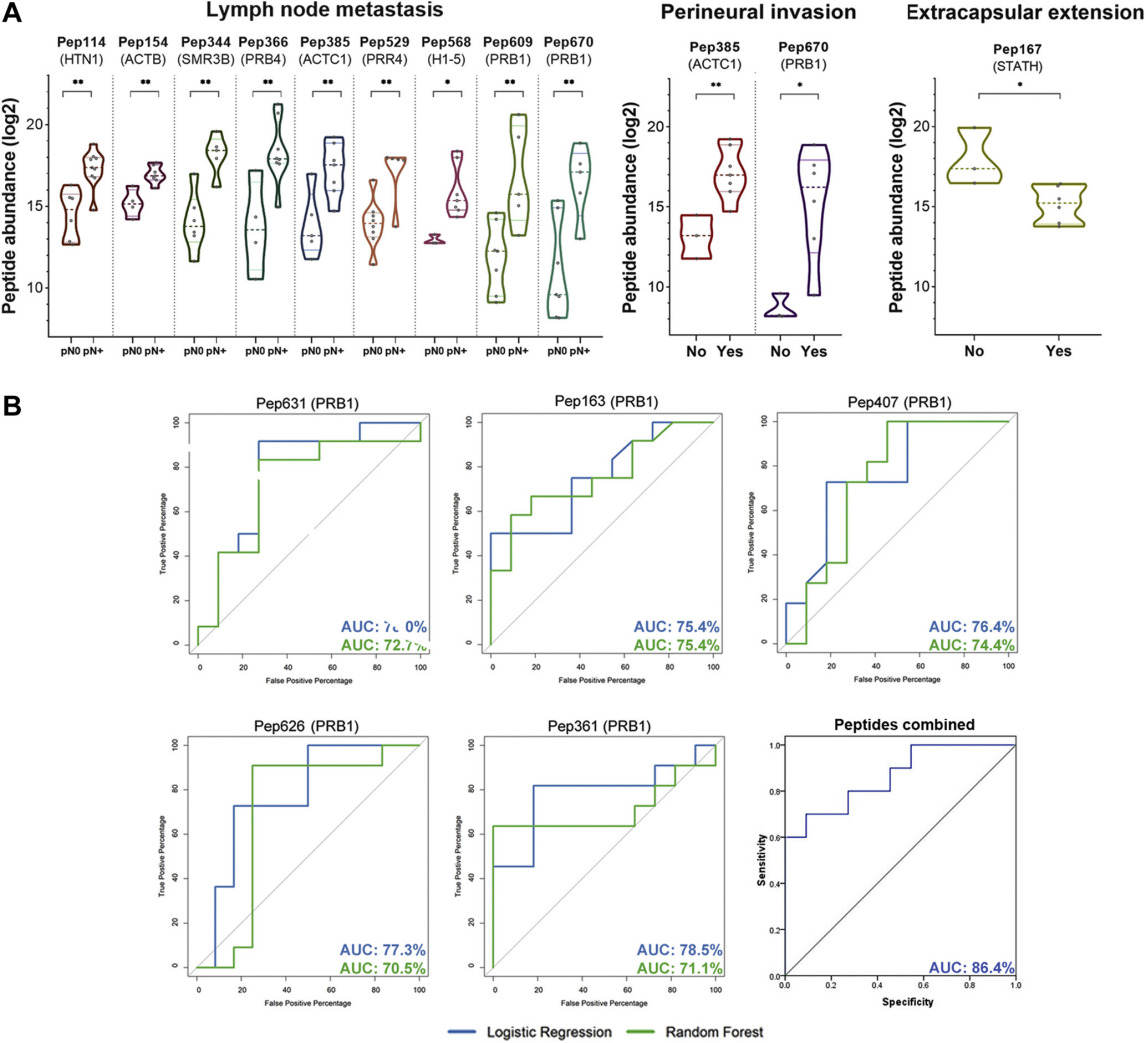
**Correlation of differential peptides with OSCC prognostic factors.***A*, ten differentially abundant peptides, with increased levels in pN+ saliva, were correlated with prognostic features such as perineural invasion, nodal metastasis, and extracapsular extension. Significance defined as ANOVA *p*-value ≤ 0.05 (∗), 0.01 (∗∗); minimum correlation coefficient +0.7/−0.7; multiple R^2^ > 0.5. *B*, in addition, top ranking peptides detected across >85% of samples were able to distinguish pN0 and pN+ patients with AUC-ROC > 0.7 calculated using logistic regression and random forest analysis. Once combined, these peptides performed better as classifiers, achieving an AUC-ROC > 0.85 by binary regression. AUC-ROC, area under the receiver operating characteristic curve; OSCC, oral squamous carcinoma.

Quantitative data from the differential peptides (pN0 *versus* pN+ comparison) were tested for association with patient's clinicopathological information with prognostic value. Initially, 25 peptides, with ≥6 valid values (∼50%) in either pN+ or pN0 two groups, presented significant association with prognostic features when default statistical thresholds were used (linear regression *p*-value < 0.05, and Pearson's R-squared > 0.5; supplemental Table S8). By applying more stringent criteria (≥3 valid values per clinical feature in addition to default thresholds), a subset of ten endogenous peptides (Pep114, Pep154, Pep167, Pep344, Pep366, Pep385, Pep529, Pep568, Pep609, Pep670) correlated with extracapsular extension and perineural invasion, in addition to lymph-node metastasis (Fig. 4*A*; supplemental Table S8). Notably, all these peptides exhibited higher abundance in pN+ saliva than in pN0. Furthermore, correlation analyses pointed out that increased levels were associated with worse prognostic factors, such as the presence of perineural invasion or nodal metastasis. Conversely, the occurrence of extracapsular extension in lymph nodes—a feature presented only in pN+ patients—was inversely proportional to Pep167 levels.

ROC curves were used to evaluate the power of differentially abundant peptides to distinguish pN0 and pN+ patients based on their quantitative profiles (supplemental Table S9). Five peptides derived from basic salivary proline-rich proteins (PRPs) (Pep631, Pep163, Pep361, Pep407, Pep626) were consistently detected in at least 85% of the group samples and exhibited an AUC-ROC higher than 70% in both random forest and logistic regression analyses (Fig. 4*B*). Interestingly, the power to classify pN0 and pN+ patients was increased to an AUC of 86% when those five peptides were combined (Fig. 4*B*), demonstrating that a molecular panel can perform better in the classification of patients with nodal metastasis.

### Cleavage Site Analysis Suggests Putative Proteases Are Able to Modulate the Salivary Peptidome

**Fig. 5:**
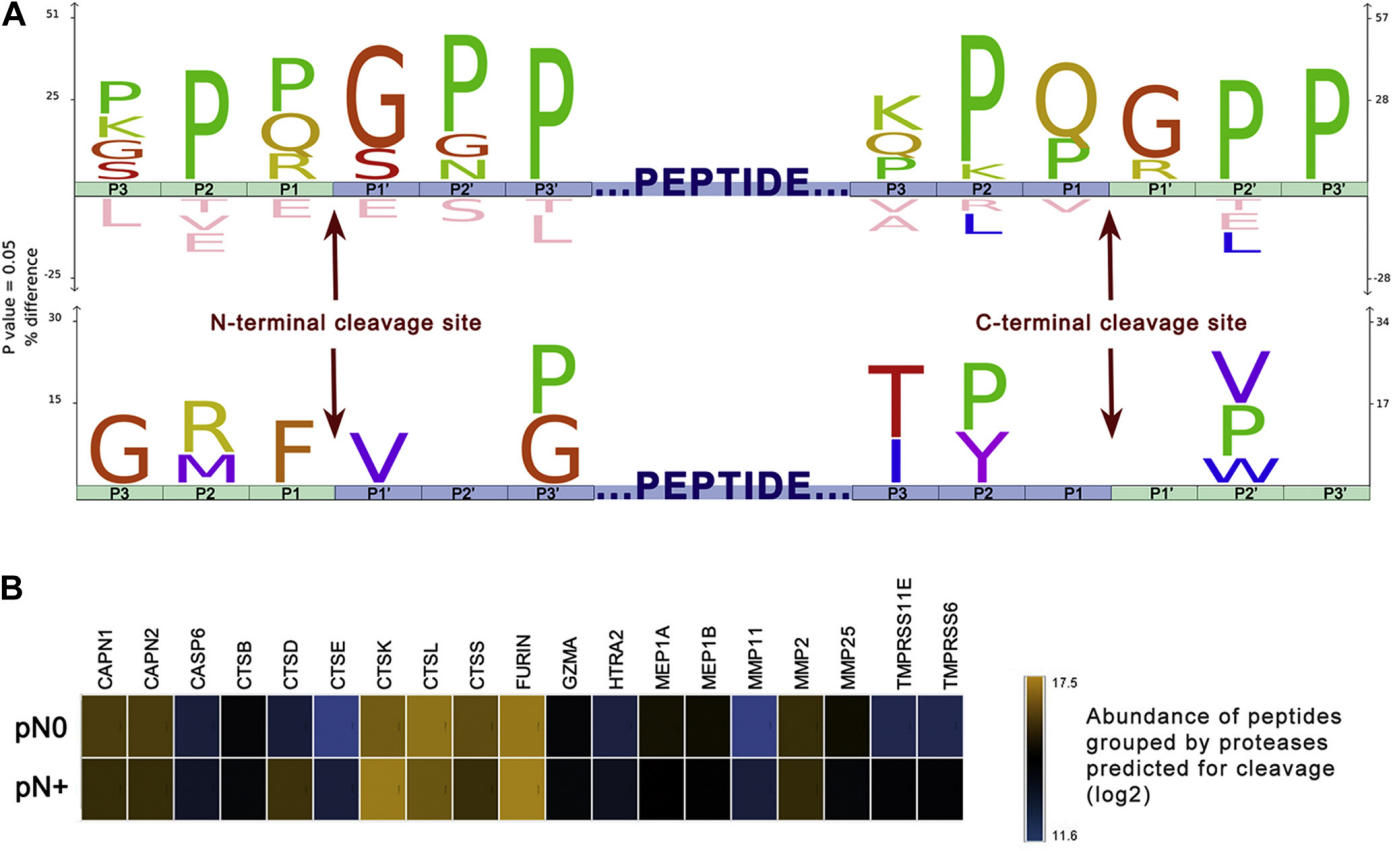
**Cleavage site analysis of differentially abundant peptides and prediction of active proteases.***A* IceLogo and heat map indicating position-specific amino acid residues under-represented and over-represented in the putative cleavage site of endogenous peptides. Three N- and C-terminal residues (depicted by *green blocks*) were derived from human protein sequences to reconstruct the putative sites cleaved (P1-P1ʹ) for releasing the differential endogenous peptides (represented by the *central blue block*). To minimize compositional bias toward the proline occurrence evidenced on the upper diagram, salivary proline-rich proteins (P04280-PRB1, Q04118-PRB3, P02812-PRB2, P10163-PRB4, P02814-SMR3B) were excluded in a second analysis (bottom diagram). *B*, LFQ abundances of differential peptides predicted as substrates of 19 proteases using Proteasix is illustrated as the heat map. Notably, proteolytic products of cathepsins K, L, and S, furin, and calpains are among the most abundant endogenous peptides. In addition, the levels of cleavage products resulting of cathepsin D (CSTD) activity exhibited higher intensities in pN+ saliva than in that of pN0.

Peptidases possibly implicated in the endogenous cleavage of saliva proteins were predicted to provide a better understanding of the proteolytic events linked to the peptidome changes observed between pN0 and pN+ samples. This was achieved by reconstructing the putative cleavage sites deriving three N- and C-terminal amino acid residues from the full-length protein sequences (supplemental Table S10). A graphical representation by the IceLogo and heat map showed a significant over-representation of peptides produced upon N-terminal cleavage between Pro/Gln/Arg and Gly/Ser, and C-terminal cleavage between Gln/Pro and Gly/Arg residues (Fig. 5*A*, top sequence logo). Notably, the enrichment of proline residues in the vicinity of the cleaved peptide bonds was recurrent because sequences derived from PRPs (UniProt IDs P04280, Q04118, P02812, P10163, P02814) composed a major fraction of our peptide list. To minimize this caveat, PRPs were filtered out and the data were reanalyzed, revealing that preferential N-terminal cleavages of non-PRPs happened between Phe and Val residues, while no C-terminal cleavage site was found enriched (Fig. 5*A*; bottom sequence logo).

Proteasix analysis has shown that 22 of the 77 differentially abundant peptides are known substrates of up to 19 specific endopeptidases and exopeptidases (supplemental Table S11) that might be affecting the saliva peptidome in a disease-specific manner. A heat map consolidating the abundance of all peptides putatively produced by the predicted proteases highlighted cathepsin K, L, and S; furin; and calpain 1 and 2 as responsible for the cleavage of the most abundant peptides (Fig. 5*B*). Overall abundance of substrates of each predicted peptidase was similar between pN0 and pN+, with the exception of peptides cleaved by cathepsin D, which presented higher intensities in N+ saliva (Fig. 5*B*).

**Fig. 6:**
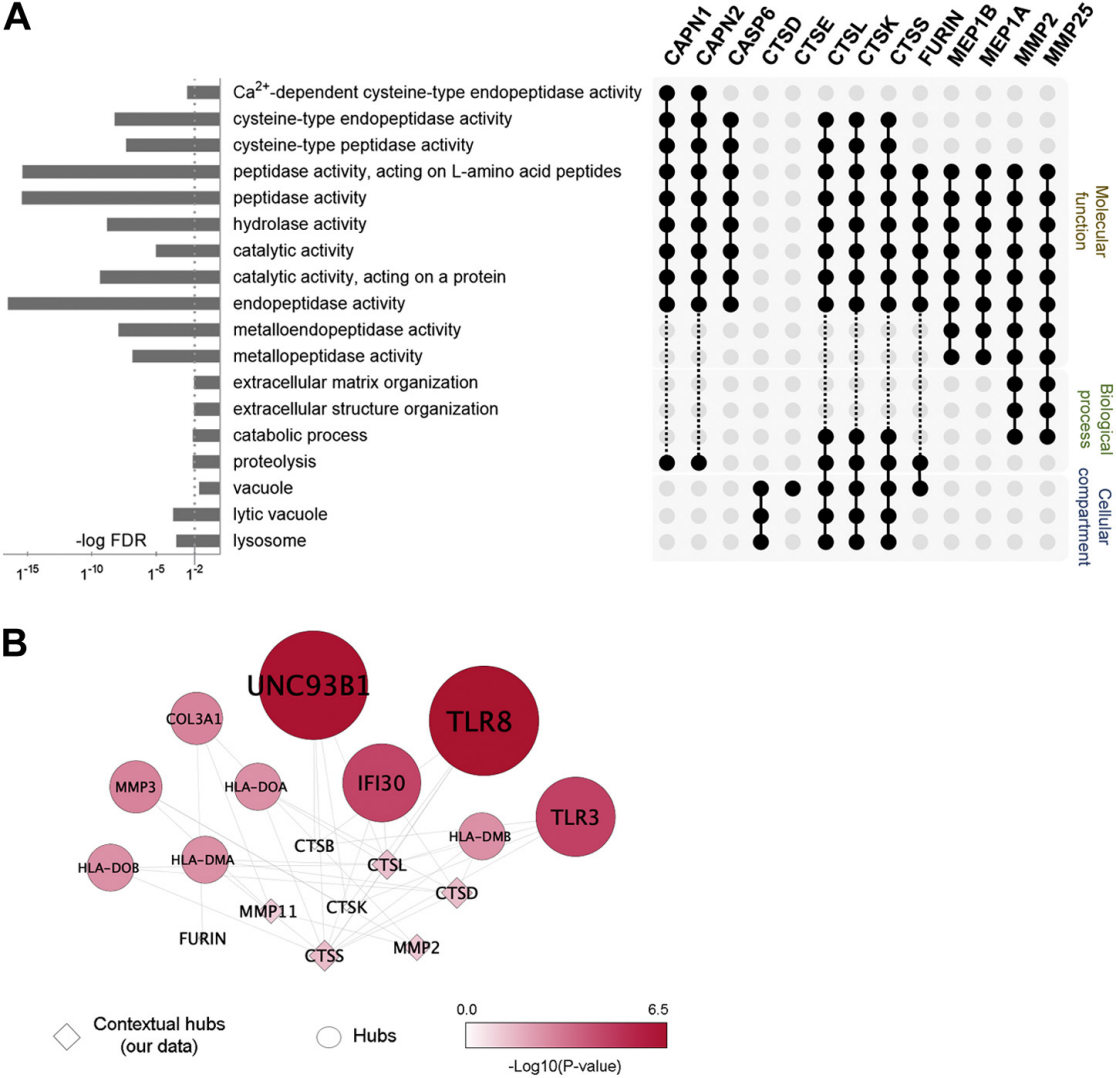
**Functional characterization of predicted proteases implicated in the processing of differential peptides.***A*, GO enrichment analysis of the predicted proteases showed a major contribution of lysosomal and vacuole cathepsins. Together, CTSL, CTSK, CTSS, CAPN1, CAPN2, and CASP6 exhibit cysteine-type peptidase activity, and membrane metalloproteinases (MMP2 and MMP25) are mostly involved in structural organization of the extracellular matrix via proteolysis. *B*, network analysis highlighted that predicted cathepsins and membrane metalloproteases are linked to immunity via Toll-like receptors and antigen presentation. Interaction hub of predicted proteases (squares) and their top ten interactors (*circles*); significance threshold *p*-value was < 0.05. GO, Gene Ontology.

GO enrichment analysis of the predicted proteases showed the over-representation of cathepsins (CTS) with lysosomal (CTSD, L, K, S) or vacuole (CTSE) origin (Fig. 6*A*). Together, cathepsins L, K, and S; calpain 1 and 2; and caspase 6 exhibit cysteine-type peptidase activity. Membrane metalloproteinase 2 and 25 were mostly involved in structural organization of the extracellular matrix via proteolysis. Network analysis highlighted that predicted cathepsins and membrane metalloproteases are linked to immunity via Toll-like receptors and antigen presentation (Fig. 6*B*).

### Expression Patterns of Predicted Proteases Are Associated With Cancer Prognosis

Because an association between differential peptides and prognostic features was observed before, we also investigated whether the expression levels of the predicted proteases could be similarly associated with cancer prognosis (supplemental Fig. S1). Publicly available PROGgene tool and gene expression data from patients with head and neck carcinoma indicated that an augmented expression of *CAPN1*, *CAPN2*, *CTSB*, and *MMP11* genes in tumor tissue is associated with reduced overall survival (<40% in 5 years), while reduced levels of *MMP25* transcripts lower 5-year overall survival. In addition, higher *CAPN1* expression also correlates with reduced metastasis-free survival, whereas relapse-free survival is diminished when *MMP25* and *TMPRSS6* expression is decreased.

Transcript levels of the predicted proteases could also be associated with specific prognostic features such as the tumor size, recurrence, invasiveness, nodal metastasis, and survival (Table 1; supplemental Fig. S2). For instance, upregulation of most cathepsins, *CAPN1* and *2*, *MEP1A*, and *MMP25*, exhibited a major correlation with poor prognostic features such as recurrence, perineural invasion, nodal extracapsular extension, and tumor size. Conversely, expression of *MMP2* and *MMP11* was found to be reduced in cases with advanced tumor stages (T3–T4) and nodal metastasis (N+).

**Table 1:** Correlation between peptidases expression and prognostic features on TCGA clinical data

Protease	Prognostic feature	*p*-value^a^	Expression pattern
CTSD	Vital status	0.009	Alive (↑UP); dead (↓DOWN)
	Recurrence	0.045	New tumor event detected (↑UP)
CTSS	Recurrence	0.044	New tumor event detected (↑UP)
	Perineural invasion	0.021	Perineural invasion present (↑UP)
CTSE	Pathologic T group	0.026	T3-T4 (↑UP); T1-T2 (↓DOWN)
CTSK	Pathologic T group	0.011	T3-T4 (↓DOWN); T1-T2 (↑UP)
CAPN1	Recurrence	0.045	New tumor event detected (↑UP)
CAPN2	Recurrence	0.003	New tumor event detected (↑UP)
	Perineural invasion	0.048	Perineural invasion present (↑UP)
	Extracapsular extension	0.011	Nodal extracapsular spread (↑UP)
MEP1A	Recurrence	0.046	New tumor event detected (↑UP)
	Pathologic T group	0.022	T3-T4 (↑UP); T1-T2 (↓DOWN)
MMP2	Pathologic T group	0.006	T3-T4 (↓DOWN); T1-T2 (↑UP)
MMP11	N status 1	0.014	N0 (↑UP); N+ (↓DOWN)
	Pathologic T group	0.009	T3-T4 (↓DOWN); T1-T2 (↑UP)
MMP25	Perineural invasion	0.046	Perineural invasion present (↑UP)

TCGA, The Cancer Genome Atlas.

The number of samples for each correlation analysis is available in supplemental Figure S2.

a The normality of gene expression data was assessed with the Shapiro-Wilk test (α = 0.05). Student's *t*-test or Welch's test (two group comparisons) and ANOVA with post hoc Tukey test (>2 groups) were used for parametric data. Nonparametric data were analyzed with Wilcoxon (two groups) or Kruskal–Wallis (>2 groups) tests. Significance threshold *p*-value was ≤0.05.

### Higher Proteolysis in Saliva May Be Associated With Diminished Inhibitory Function

**Fig. 7:**
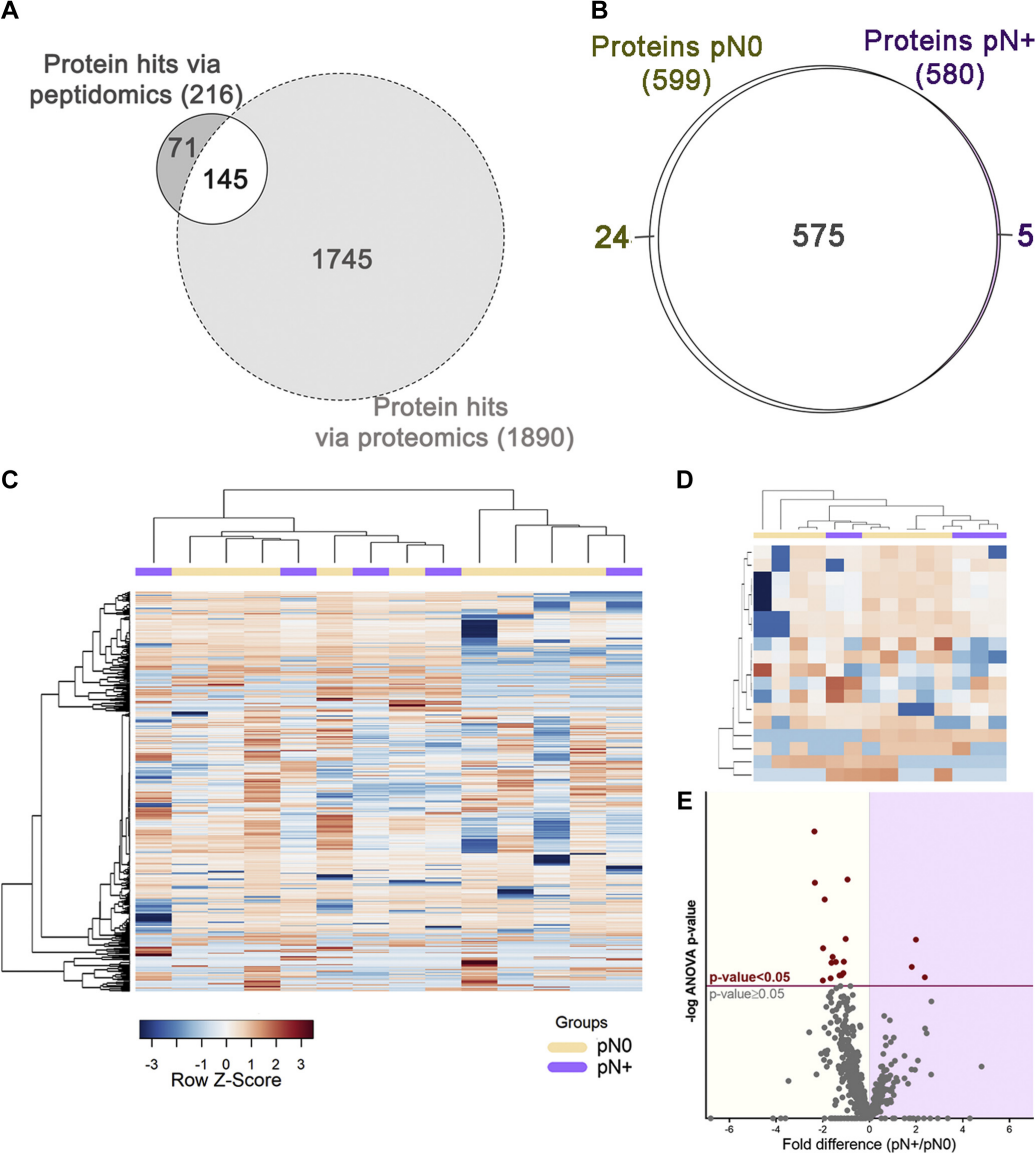
**Composition of the saliva proteome from pN0 and pN+ OSCC patients.***A*, the Venn diagram of saliva proteins exclusive or commonly identified on peptidomics and proteomics approaches. *B*, qualitative analysis reveals a major overlap between N0 and N+ saliva proteomes. *C*, the heat map of the whole saliva proteome and (*D*) differentially abundant proteins (ANOVA *p*-value ≤ 0.05) show no clear grouping of samples based on a single classifier feature like lymph-node metastasis (N+/N0). Hierarchical clustering was performed using the Pearson's correlation and Ward's method. *E*, differential distribution of proteins quantified in pN0 and pN+ samples represented on a volcano plot. Proteins above the significance threshold (ANOVA *p*-value ≤ 0.05) are highlighted as *red dots*.

Bottom-up proteomics was applied to characterize the saliva proteome using a second cohort of pN0 and pN+ patients. This analysis provided valuable information on the levels of saliva proteases, protease inhibitors, and protein substrates prone to proteolysis. Most saliva proteins previously identified through peptide fragments released by endogenous proteolysis (peptidomics) were also detected using bottom-up proteomics (Fig. 7*A*). Interestingly, none of those exhibited significant changes between the pN0 and pN+ saliva (supplemental Tables S12 and S13), indicating that changes in the proteolysis balance detected via peptidomics are off the radar of conventional bottom-up proteomics.

**Fig. 8:**
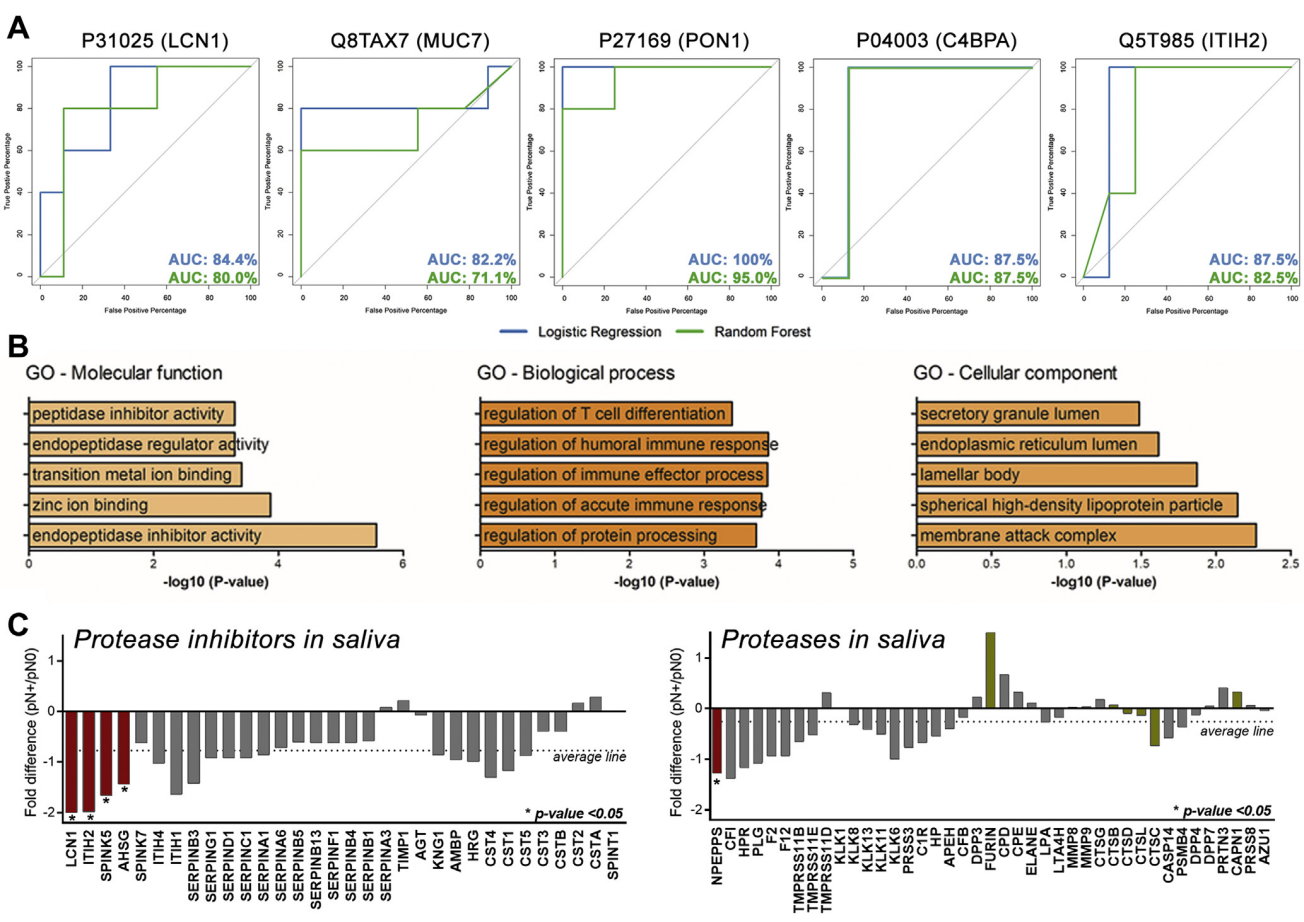
**Functional analysis of differentially abundant proteins and ROC curves.***A*, ROC curves of top-ranking peptides detected across >85% of samples exhibited AUC-ROC > 0.8, calculated using logistic regression and random forest analysis. *B*, GO analysis performed by Enrichr highlights peptidase inhibitory activity and immune-related processes among differentially abundant proteins in saliva. *C*, quantitative profiles showed a −0.8-fold (log2) reduction on average abundance of peptidase inhibitors in pN+ saliva. Differences on LCN1, ITIH2, SPINK5, and AHSG protein levels were statistically significant (ANOVA *p*-value ≤ 0.05; *red bar*). On the other hand, average differences in protease levels were less pronounced (−0.2-fold log2 pN+/pN0) with a single peptidase - NPEPPS, puromycin-sensitive amino peptidase - differentially abundant (ANOVA *p*-value ≤ 0.05; *red bar*). Predicted proteases implicated in the processing of differential endogenous peptides are indicated by *golden bars*. AUC-ROC, area under the receiver operating characteristic curve; GO, Gene Ontology.

Notably, group variation in saliva proteome was less remarkable when compared with the peptidome component, showing only 5 and 24 proteins exclusively detected in pN+ and pN0 groups, respectively (Fig. 7*B*). Similar to what was observed in the peptidomic analysis, group-specific differences at the protein level did not result in the perfect separation of pN+ and pN0 patients, as represented by the hierarchical clustering heat maps of the whole proteome and differential proteins (Fig. 7, *C*–*D*). The volcano plot indicates 18 proteins detected at different levels between the two groups (ANOVA *p*-value ≤ 0.05; Fig. 7*E*). Of these, a subset comprising lipocalin-1 (LCN1), mucin-7 (MUC7), serum paraoxonase/arylesterase-1 (PON1), C4b-binding protein alpha chain (C4BPA), and inter-alpha-trypsin inhibitor heavy chain H2 (ITIH2) has proved to be useful classifiers of pN0 and pN+ cases by displaying AUC-ROC of logistic regression >80% (Fig. 8*A*, supplemetal Table S14). Moreover, among all clinicopathological features tested, only lymph node metastasis was found correlated with differentially abundant proteins (supplemental Fig. S3).

GO analysis of the 18 differentially abundant saliva proteins revealed the enrichment of terms related to peptidase/endopeptidase inhibitor activities (Fig. 8*B*). More specifically, we found that 32 protease inhibitors quantified in saliva exhibited an −0.8× (log2) average reduction in N+ patients' saliva, whereas fetuin A (AHSG), ITIH2, LCN1, and SPINK5 revealed statistically relevant changes (ANOVA *p*-value ≤ 0.05; Fig. 8*C*). Interestingly, in over 40 proteases quantified across the samples, only puromycin-sensitive aminopeptidase (NPEPPS) displayed significant changes between pN0 and pN+ patients (ANOVA *p*-value ≤ 0.05), while the average difference between the groups was closer to the equivalence (average −0.2 × log2; Fig. 8*C*).

**Fig. 9:**
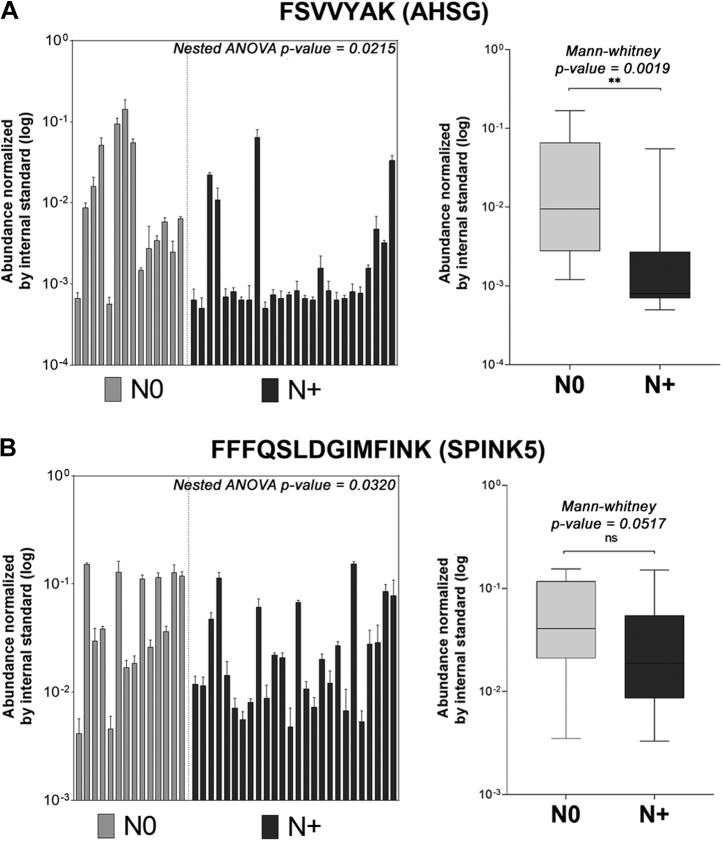
**Exploratory SRM-MS analysis of protease inhibitor levels in saliva.***A*, levels of AHSG inferred by FSVVYAK peptide across an independent 40-patient cohort. AHSG was significantly reduced in N+ saliva, with minor intragroup variation (nested ANOVA, *p*-value = 0.0215) and significant difference on group medians (Mann–Whitney, *p*-value = 0.0019). *B*, saliva levels of SPINK5 inferred via FFFQSLDGIMFINK peptide exhibited a higher intragroup variation affecting the confirmation of group differences (Mann–Whitney, *p*-value > 0.05). AHSG, alpha-2-HS-glycoprotein (fetuin A); SPINK5, serine protease inhibitor Kazal-type 5; SRM-MS, selected reaction monitoring–mass spectrometry. ANOVA *p*-value > 0.05 (ns), ≤0.05 (∗), ≤0.01 (∗∗).

The reduced levels of fetuin A (AHSG) and SPINK5 on N+ saliva was confirmed by a targeted SRM-MS assay using 40 samples of OSCC saliva (N0, *n* = 14; N+, *n* = 26). More specifically, fetuin A proteotypic peptide FSVVYAK revealed a significant group difference with minor intragroup variations (Fig. 9*A*). Of note, a second peptide HTLNQIDEVK was also monitored for fetuin A but the lack of consistent signal of the endogenous peptide (light channel) indicated that the analyte was below the limit of detection (Supplemental Fig. S4). Protein levels of SPINK5 inferred from the FFQSLDGIMFINK peptide also suggested lower levels in N+ saliva, although a high variation among individuals was observed, thus affecting the verification of statistically significant group differences (Fig. 9*B*). Two other peptides selected for SPINK5 quantification exhibited low ion response and were not detected in our SRM analysis (supplemental Fig. S5). Together these data indicated that reduced levels of protease inhibitors in patients with lymph node metastasis concur with the accentuated proteolysis observed in the same group.

## Discussion

We have developed a peptide-driven workflow able to assist a molecular-based prognosis of OSCC via peptidomics and proteomics. The use of saliva has been proposed because it can be easily collected via noninvasive methodology and it is in contact with the cancer lesion. The potential of saliva for providing potential OSCC biomarkers at the protein level using traditional bottom-up proteomics has been demonstrated by our group (16, 36). However, saliva is a rich resource of peptides derived from endogenous proteolysis of soluble constituents. Compared with other body fluids with clinical relevance (*e.g.*, blood, urine, cerebrospinal fluid), low-molecular-weight peptides account for a higher fraction (∼30%) of total proteinaceous content of saliva (37), what makes the recovery of small peptides easier and faster than conventional bottom-up proteomic analyses.

A variety of approaches, including the use of organic solvents (*e.g.*, methanol, MeCN), acids (TFA, HCl), chaotropic agent (urea), ultrafiltration spin devices, and chromatography, have been shown to be feasible in the extraction of endogenous peptides from complex matrixes (37). Different methods can bias the extraction toward intrinsic physicochemical features of peptides likely affecting the yield and purity or even leading to the enrichment of structurally modified bioactive peptides (38). Here we evaluated the efficiency of two methods of extraction and three chromatography settings in achieving robust and fast characterization of the saliva peptidome in patients with OSCC. Our extraction methods explored peptide hydrophobicity upon C_18_ solid-phase acidic extraction (HCl-SPE) or separating smaller peptides from proteins based on molecular ultrafiltration under denaturing conditions (4 M urea), followed by C_18_ reversed-phase desalting (UF-SPE).

The HCl-SPE method demonstrated increased peptidome coverage and identification rates with no compromise to the recovery of long peptides, whereas the UF-SPE method filtered out molecules longer than 27 to 30 residues, likely explained by the molecular weight cut-off of 3 kDa. Although membranes with larger pore sizes could be alternatively used, this might result in a peptidome significantly more contaminated with intact low-molecular-weight proteins because a large proportion (∼20%) of the saliva proteome is composed of proteins <20 kDa (39). Importantly, search engines often have a maximum peptide length set to ∼30 residues as default, which might affect identification rates of longer peptides and hence should be adjusted accordingly.

The optimized workflow developed in this work was successfully applied in the characterization of the salivary peptidome from OSCC patients with or without lymph node metastasis. Interestingly, the higher number of endogenous peptides identified exclusively in N+ patients, together with the increased peptide abundances in the same group, suggests that proteolysis is accentuated in individuals with lymph node metastasis. In fact, the proteolytic activity in saliva has been reported to be increased in patients with OSCC compared with that in healthy individuals or in those with other benign oral conditions (40).

Of note, our results suggested that proteolysis not only is accentuated in OSCC but also can be regulated over the clinical course of the disease. However, we noticed that quantitative information from either the whole saliva peptidome/proteome or differential peptides/proteins was not able to perfectly group pN0 and pN+ patients in the hierarchical clustering. This suggested that other factors might be determinant in the saliva composition, thus affecting the performance of the lymph node metastasis status as a single variable. In fact, at least in the peptidomic analysis, the results of the correlation analysis reinforced that the quantitative pattern of differential molecules is also associated with pathological features other than lymph node metastasis, such as perineural invasion and extracapsular extension.

At least five endogenous peptides derived from basic salivary proline-rich protein 1, LCN1, MUC7, serum PON1, C4BPA, and ITIH2 proteins displayed the ability to better distinguish pN0 and pN+ patients. Therefore, our results demonstrated that even with the compositional variation inherent in the saliva samples, a molecular panel to assess lymph node metastasis status could be established. Peptidomics patterns have been previously reported as capable of discriminating cancer types, such as breast, prostate, and bladder (41). Previous work has shown that breast cancer plasma peptidomics profiles can be strikingly different between the patients with early-stage breast cancer and healthy controls (8), providing its application value besides conventional bottom-up proteomics.

Information regarding proteolytic activity could be anticipated by peptidomics analysis and protease predictions. The remarkable enrichment of proline residues in the sequence logo highlighted a major contribution of breakdown products derived from salivary PRP in the saliva of pN+ patients. We acknowledge, together with histatins and statherins, PRP fragments as the most abundant products of in-mouth proteolysis (42). PRP peptides are often released upon cleavage of the Gln-Gly (P1-P1ʹ) motif (43), which was found enriched in both N- and C- terminals of differential peptides, likely as result of a glutamine endopeptidase with microbial origin (44). Importantly, the role of oral microbiota in cancer development (45) and OSCC aggressiveness (46) has been recently addressed, reinforcing that oral microbiome can be intimately linked to molecular aspects of OSCC biology. Noteworthy, four of five differential peptides with the highest AUC-ROC (Pep631, Pep163, Pep407, Pep361) were derived from basic salivary PRPs upon cleavage of the Gln-Gly motif. Notably, our data suggest that the intrinsic presence and activity of microbe contaminants in whole saliva provide unique features relevant for patient classification. However, the collection of less-contaminated fluids, such as parotid saliva, can also be considered in OSCC studies.

When PRPs are not considered in the sequence logo analysis, an additional cleavage specificity highlighted hydrolysis after phenylalanine residues. Cathepsin D is known to cleave at the C-term of aromatic residues and plays a major role in the oral cavity proteolysis by promoting the breakdown of histatins and statherins (47). Interestingly, among all proteases predicted, the cleavage products related to cathepsin D were notably more abundant in pN+ saliva, suggesting an increased activity in the saliva of patients with OSCC and lymph-node metastasis. The relationship between tissue levels of cathepsin D and invasion, progression, and metastasis has been observed for oral carcinoma (48, 49). Although it is primarily located within lysosomes in physiological conditions, *CTSD* overexpression in cancer cells results in hypersecretion of this protease (50), which might eventually lead to its increased activity in saliva. Similarly, other predicted cathepsins also exhibit vital intracellular roles. As anticipated by the network analysis, the cysteine cathepsins B, K, and L are linked to immune-related processes via Toll-like receptors and human leukocyte antigen associated with major histocompatibility complex class II (MHC class II), thus highlighting that imbalances in their catalytic activity have the potential to impair immune responses to cancer (51).

Expression data available in public repositories suggested that increased tissue expression of calpains 1 and 2 and *MMP11* decrease overall patient survival and higher levels of meprin 1A can be associated with recurrence and tumor size. However, although mRNA levels in tumor tissues suggest a regulation on proteolytic processes via peptidase levels, this may not be entirely reflected in the saliva peptidome for many reasons. For instance, our results support the finding that the saliva peptidome is a pool of products generated by peptidases whose origin is in the oral microflora (*e.g.*, bacteria glutamine protease), host salivary glands, immune cells (*e.g.*, proteasix predicted granzyme A, GZMA), and tumor cells. Therefore, the peptidomic-driven strategy might constitute a singular approach to assess the outcome of peptidase activity in saliva, regardless of the expression patterns observed in adjacent tissues/cells.

Remarkably, enzymatic activity of peptidases can be regulated by multiple mechanisms including their own expression levels, structural modification (*e.g.*, zymogen processing and post-translational modifications), and changes in inhibitory control. As a matter of fact, our parallel bottom-up proteomic analysis showed only small differences in the levels of proteases quantified in saliva, whereas protease inhibitors exhibited an important reduction in pN+ samples. This led to the assumption that modulation of inhibitory functions may in great part induce the changes in the saliva peptidome in more advanced cases of OSCC diagnosed with lymph-node metastasis.

The four protease inhibitors significantly reduced in N+ saliva, namely, fetuin A (AHSG), ITIH2, LCN1, and SPINK5, display inhibitory activity over a wide range of serine and cysteine proteases. SPINK5 protein possesses a major inhibitory activity on kallikrein 5, which has been described with increased activity in OSCC saliva (40), whereas fetuin A (AHSG) displays a broad-range inhibitory activity including meprin zinc metalloproteinases and trypsin (52). Noteworthy, fetuin A has been proposed as a marker for metastasis in prostate cancer (53, 54).

The lower levels of fetuin A in the N+ saliva could be confirmed by SRM-MS analysis on a larger and independent cohort of patients with OSCC. These new findings indicated a similar tendency with our previous results on the cysteine–protease inhibitor, cystatin-B, that presented reduced levels in neoplastic islands from the invasive tumor front and also in the saliva of N+ OSCC patients compared with that in N0 OSCC patients (19). Of note, quantification of SPINK5 protein via targeted proteomics remains inconclusive as the only high-responding peptide detected exhibited suboptimal quantotypic qualities (*i.e.*, presence of methionine residue), which might have contributed to the higher variation observed. In addition, poor detection of the two additional SPINK5 peptides provides evidence that the selection of peptides for targeted proteomics remains challenging. In this context, the use of computational tools able to predict peptides with the highest ion response might increase the success rate (55, 56). Although the inference of protein abundances using a single peptide may not be ideal, we observed that label-free quantification using data-dependent acquisition and label-based using SRM from two independent patient cohorts corroborated the slightly reduced levels of fetuin A and SPINK5 protease inhibitors in OSCC N+ saliva.

Taken together, the strategy we presented here constitutes a multifaceted approach for comprehensive characterization of proteolytic events in OSCC via peptidomics, bottom-up proteomics, and *in silico* analysis of cleavage sites. The entire quantitative workflow, encompassing optimized sample preparation, LC-MS/MS acquisition, and data analysis, provided a basis for future peptidomics applications in biological and translational studies. We demonstrated that the salivary peptidome is differentially regulated in patients with OSCC and nodal metastasis as a result of increased activity of proteases with oral microflora or host cell origin. This accentuated proteolysis concurs with a reduction of protease inhibitors in the saliva proteome. Specific features of the proteolytic network such as the peptide fragments of basic salivary proline-rich protein 1 and the proteins LCN1, MUC7, PON1, C4BPA, ITIH2, and AHSG have proved useful in distinguishing patients with cervical lymph-node metastasis. Therefore, our results ultimately provided a panel of analytes with prognostic utility that might assist subgrouping patients with poor prognosis requiring tailored therapeutic interventions.

## Data availability

The mass spectrometry proteomics data have been deposited to the ProteomeXchange Consortium via the PRIDE partner repository (57) with the data set identifiers PXD020211 (10.6019/PXD020211) and PXD020111, annotated spectra deposited in MS viewer (58) with search key: kbc7zh9oov). The SRM analyses are available through the Panorama Public repository at the following link: https://panoramaweb.org/WirByY.url and ProteomeXchange data set PXD020237.

## Conflict of interest

The authors declare no competing interests.

## References

[1] Doucet A., Butler G.S., Rodríguez D., Prudova A., Overall C.M. (2008). Metadegradomics. Mol. Cell. Proteomics.

[2] Chang C., Werb Z. (2001). The many faces of metalloproteases: cell growth, invasion, angiogenesis and metastasis. Trends Cell Biol.

[3] Mason S.D., Joyce J.A. (2011). Proteolytic networks in cancer. Trends Cell Biol.

[4] Sevenich L., Joyce J.A. (2014). Pericellular proteolysis in cancer. Genes Dev..

[5] Deng Z., Li Y., Fan J., Wang G., Li Y., Zhang Y., Cai G., Shen H., Ferrari M., Hu T.Y. (2015). Circulating peptidome to indicate the tumor-resident proteolysis. Sci. Rep..

[6] Baker E.S., Liu T., Petyuk V.A., Burnum-Johnson K.E., Ibrahim Y.M., Anderson G.A., Smith R.D. (2012). Mass spectrometry for translational proteomics: progress and clinical implications. Genome Med..

[7] Njoku K., Chiasserini D., Whetton A.D., Crosbie E.J. (2019). Proteomic biomarkers for the detection of endometrial cancer. Cancers (Basel).

[8] Xu Z., Wu C., Xie F., Slysz G.W., Tolic N., Monroe M.E., Petyuk V.A., Payne S.H., Fujimoto G.M., Moore R.J., Fillmore T.L., Schepmoes A.A., Levine D.A., Townsend R.R., Davies S.R. (2015). Comprehensive quantitative analysis of ovarian and breast cancer tumor peptidomes. J. Proteome Res..

[9] Secher A., Kelstrup C.D., Conde-Frieboes K.W., Pyke C., Raun K., Wulff B.S., Olsen J.V. (2016). Analytic framework for peptidomics applied to large-scale neuropeptide identification. Nat. Commun..

[10] Marino G., Eckhard U., Overall C.M. (2015). Protein termini and their modifications revealed by positional proteomics. ACS Chem. Biol..

[11] Klein J., Eales J., Zürbig P., Vlahou A., Mischak H., Stevens R. (2013). Proteasix: a tool for automated and large-scale prediction of proteases involved in naturally occurring peptide generation. Proteomics.

[12] Schilling O., Overall C.M. (2008). Proteome-derived, database-searchable peptide libraries for identifying protease cleavage sites. Nat. Biotechnol..

[13] Ling X.B., Lau K., Deshpande C., Park J.L., Milojevic D., Macaubas C., Xiao C., Lopez-Avila V., Kanegaye J., Burns J.C., Cohen H., Schilling J., Mellins E.D. (2010). Urine peptidomic and targeted plasma protein analyses in the diagnosis and monitoring of systemic juvenile idiopathic arthritis. Clin. Proteomics.

[14] Bauca J.M., Martinez-Morillo E., Diamandis E.P. (2014). Peptidomics of urine and other biofluids for cancer diagnostics. Clin. Chem..

[15] Hosmer D.W., Lemeshow S., Sturdivant R.X. (2013). Applied Logistic Regression, 3rd Ed..

[16] Winck F.V., Prado Ribeiro A.C., Ramos Domingues R., Ling L.Y., Riaño-Pachón D.M., Rivera C., Brandão T.B., Gouvea A.F., Santos-Silva A.R., Coletta R.D., Paes Leme A.F. (2015). Insights into immune responses in oral cancer through proteomic analysis of saliva and salivary extracellular vesicles. Sci. Rep..

[17] Dephoure N., Gygi S.P. (2011). A solid phase extraction-based platform for rapid phosphoproteomic analysis. Methods.

[18] Rappsilber J., Mann M., Ishihama Y. (2007). Protocol for micro-purification, enrichment, pre-fractionation and storage of peptides for proteomics using StageTips. Nat. Protoc.

[19] Carnielli C.M., Macedo C.C.S., De Rossi T., Granato D.C., Rivera C., Domingues R.R., Pauletti B.A., Yokoo S., Heberle H., Busso-Lopes A.F., Cervigne N.K., Sawazaki-Calone I., Meirelles G.V., Marchi F.A., Telles G.P. (2018). Combining discovery and targeted proteomics reveals a prognostic signature in oral cancer. Nat. Commun..

[20] Zhang J., Xin L., Shan B., Chen W., Xie M., Yuen D., Zhang W., Zhang Z., Lajoie G.A., Ma B. (2012). PEAKS DB: de novo sequencing assisted database search for sensitive and accurate peptide identification. Mol. Cell. Proteomics.

[21] Choi H., Ghosh D., Nesvizhskii A.I. (2008). Statistical validation of peptide identifications in large-scale proteomics using the target-decoy database search strategy and flexible mixture modeling. J. Proteome Res..

[22] Liaw A., Wiener M. (2002). Classification and regression by randomForest. R. News.

[23] Robin X., Turck N., Hainard A., Tiberti N., Lisacek F., Sanchez J.-C., Müller M. (2011). pROC: an open-source package for R and S+ to analyze and compare ROC curves. BMC Bioinformatics.

[24] Martens L., Vandekerckhove J., Gevaert K. (2005). DBToolkit: processing protein databases for peptide-centric proteomics. Bioinformatics.

[25] Huang D.W., Sherman B.T., Lempicki R.A. (2009). Systematic and integrative analysis of large gene lists using DAVID bioinformatics resources. Nat. Protoc..

[26] Muetze T., Goenawan I.H., Wiencko H.L., Bernal-Llinares M., Bryan K., Lynn D.J. (2016). Contextual Hub Analysis Tool (CHAT): a Cytoscape app for identifying contextually relevant hubs in biological networks. F1000Research.

[27] Goswami C.P., Nakshatri H. (2014). PROGgeneV2: enhancements on the existing database. BMC Cancer.

[28] Wichmann G., Rosolowski M., Krohn K., Kreuz M., Boehm A., Reiche A., Scharrer U., Halama D., Bertolini J., Bauer U., Holzinger D., Pawlita M., Hess J., Engel C., Hasenclever D. (2015). The role of HPV RNA transcription, immune response-related gene expression and disruptive TP53 mutations in diagnostic and prognostic profiling of head and neck cancer. Int. J. Cancer.

[29] Fountzilas E., Kotoula V., Angouridakis N., Karasmanis I., Wirtz R.M., Eleftheraki A.G., Veltrup E., Markou K., Nikolaou A., Pectasides D., Fountzilas G. (2013). Identification and validation of a multigene predictor of recurrence in primary laryngeal cancer. PLoS One.

[30] Jung A.C., Job S., Ledrappier S., Macabre C., Abecassis J., de Reyniès A., Wasylyk B. (2013). A poor prognosis subtype of HNSCC is consistently observed across methylome, transcriptome, and miRNome analysis. Clin. Cancer Res..

[31] Yeung K.Y., Fraley C., Murua A., Raftery A.E., Ruzzo W.L. (2001). Model-based clustering and data transformations for gene expression data. Bioinformatics.

[32] Cox D.R. (1972). Regression models and life-tables. J. R. Stat. Soc. Ser. B.

[33] Lange V., Picotti P., Domon B., Aebersold R. (2008). Selected reaction monitoring for quantitative proteomics: a tutorial. Mol. Syst. Biol..

[34] Gallien S., Duriez E., Domon B. (2011). Selected reaction monitoring applied to proteomics. J. Mass Spectrom..

[35] Team R.C. (2013). R: a Language and Environment for Statistical Computing.

[36] Kawahara R., Bollinger J.G., Rivera C., Ribeiro A.C.P., Brandão T.B., Leme A.F.P., MacCoss M.J. (2016). A targeted proteomic strategy for the measurement of oral cancer candidate biomarkers in human saliva. Proteomics.

[37] Vitorino R., Ferreira R., Caseiro A., Amado F. (2014). Salivary peptidomics targeting clinical applications. Compr. Anal. Chem..

[38] Fleites L.A., Johnson R., Kruse A.R., Nachman R.J., Hall D.G., MacCoss M.J., Heck M. (2020). Peptidomics approaches for the identification of bioactive molecules from Diaphorina citri. J. Proteome Res..

[39] Yan W., Apweiler R., Balgley B.M., Boontheung P., Bundy J.L., Cargile B.J., Cole S., Fang X., Gonzalez-Begne M., Griffin T.J., Hagen F., Hu S., Wolinsky L.E., Lee C.S., Malamud D. (2009). Systematic comparison of the human saliva and plasma proteomes. Proteomics Clin. Appl..

[40] Feng Y., Li Q., Chen J., Yi P., Xu X., Fan Y., Cui B., Yu Y., Li X., Du Y., Chen Q., Zhang L., Jiang J., Zhou X., Zhang P. (2019). Salivary protease spectrum biomarkers of oral cancer. Int. J. Oral Sci..

[41] Liu T., Rodland K.D., Smith R.D. (2018). Characterization of the ovarian tumor peptidome. Vitam. Horm..

[42] Amado F.M.L., Ferreira R.P., Vitorino R. (2013). One decade of salivary proteomics: current approaches and outstanding challenges. Clin. Biochem..

[43] Amado F., Lobo M.J.C., Domingues P., Duarte J.A., Vitorino R. (2010). Salivary peptidomics. Expert Rev. Proteomics.

[44] Thomadaki K., Helmerhorst E.J., Tian N., Sun X., Siqueira W.L., Walt D.R., Oppenheim F.G. (2011). Whole-saliva proteolysis and its impact on salivary diagnostics. J. Dent. Res..

[45] Zhang W., Wang S., Wang H., Tang Y.-J., Tang Y., Liang X. (2019). Who is who in oral cancer?. Exp. Cell Res..

[46] Kamarajan P., Ateia I., Shin J.M., Fenno J.C., Le C., Zhan L., Chang A., Darveau R., Kapila Y.L. (2020). Periodontal pathogens promote cancer aggressivity via TLR/MyD88 triggered activation of Integrin/FAK signaling that is therapeutically reversible by a probiotic bacteriocin. PLOS Pathog..

[47] Vitorino R., Barros A., Caseiro A., Domingues P., Duarte J., Amado F. (2009). Towards defining the whole salivary peptidome. Proteomics Clin. Appl..

[48] G K., Y K., A M. (2002). Cathepsin expression in oral squamous cell carcinoma: relationship with clinicopathologic factors. Oral Surg. Oral Med. Oral Pathol. Oral Radiol. Endod..

[49] Kapoor S., Kaur G.P., Sikka P. (2014). Detection of oral squamous cell carcinoma metastasis with cathepsin D: an immunohistochemical approach. Dent. Res. J. (Isfahan)..

[50] Masson O., Bach A.-S., Derocq D., Prébois C., Laurent-Matha V., Pattingre S., Liaudet-Coopman E. (2010). Pathophysiological functions of cathepsin D: targeting its catalytic activity versus its protein binding activity?. Biochimie.

[51] Jakoš T., Pišlar A., Jewett A., Kos J. (2019). Cysteine cathepsins in tumor-associated immune cells. Front. Immunol..

[52] Hedrich J., Lottaz D., Meyer K., Yiallouros I., Jahnen-Dechent W., Stöcker W., Becker-Pauly C. (2010). Fetuin-A and cystatin C are endogenous inhibitors of human meprin metalloproteases. Biochemistry.

[53] Mintz P.J., Rietz A.C., Cardó-Vila M., Ozawa M.G., Dondossola E., Do K.-A., Kim J., Troncoso P., Logothetis C.J., Sidman R.L., Pasqualini R., Arap W. (2015). Discovery and horizontal follow-up of an autoantibody signature in human prostate cancer. Proc. Natl. Acad. Sci. U. S. A..

[54] Stone L. (2015). Prostate cancer: fetuin-A--a marker for metastatic disease?. Nat. Rev. Urol..

[55] Eyers C.E., Lawless C., Wedge D.C., Lau K.W., Gaskell S.J., Hubbard S.J. (2011). CONSeQuence: prediction of reference peptides for absolute quantitative proteomics using consensus machine learning approaches. Mol. Cell Proteomics.

[56] Fusaro V.A., Mani D.R., Mesirov J.P., Carr S.A. (2009). Prediction of high-responding peptides for targeted protein assays by mass spectrometry. Nat. Biotechnol..

[57] Vizcaíno J.A., Côté R.G., Csordas A., Dianes J.A., Fabregat A., Foster J.M., Griss J., Alpi E., Birim M., Contell J., O'Kelly G., Schoenegger A., Ovelleiro D., Pérez-Riverol Y., Reisinger F. (2012). The Proteomics Identifications (PRIDE) database and associated tools: status in 2013. Nucleic Acids Res..

[58] Baker P.R., Chalkley R.J. (2014). MS-Viewer: a web-based spectral viewer for proteomics results. Mol. Cell. Proteomics.

