# Supplementary material for: Peptidomics-Driven Strategy Reveals Peptides and Predicted Proteases Associated With Oral Cancer Prognosis
Source: Mol Cell Proteomics. 2020 Dec 3;20:100004. doi: 10.1074/mcp.RA120.002227 (PMC7950089; doi:10.1074/mcp.RA120.002227)

SUPPLEMENTARY FIGURES

Peptidomics-driven strategy reveals peptides and predicted proteases associated with oral cancer prognosis

Leandro Xavier Neves[1], Daniela Campos Granato[1], Ariane Fidelis Busso Lopes[1], Carolina M. Carnielli[1], Fábio M. de Sá Patroni[2], Tatiane De Rossi[1], Ana Karina Oliveira[1], Ana Carolina Prado-Ribeiro[3], Thais Bianca Brandão[3], André Nimtz Rodrigues[4], Pammela Araujo Lacerda[5], Miyuki Uno[6], Nilva Cervigne[5], Alan Roger Santos-Silva[7], Luiz Paulo Kowalski[8], Marcio Ajudarte Lopes[7], Adriana Franco Paes Leme*[1]

[1]Brazilian Biosciences National Laboratory, National Center for Research in Energy and Materials, Campinas, Brazil; [2]Molecular Biology and Genetic Engineering Center, University of Campinas, Campinas, Brazil; [3]Dental Oncology Service, São Paulo Cancer Institute, São Paulo, Brazil; [4]Department of Head and Neck Surgery, Faculty of Medicine of Jundiaí, Jundiaí, Brazil; [5]Department of Internal Medicine, Molecular Biology and Cell Culture Laboratory, Faculty of Medicine of Jundiaí, Jundiaí, Brazil; [6]Center for Translational Research in Oncology, São Paulo Cancer Institute, São Paulo, Brazil; [7]Oral Diagnosis Department, Piracicaba Dental School, University of Campinas, Piracicaba, São Paulo, Brazil;[8]Head and Neck Surgery, Faculty of Medicine, University of São Paulo, São Paulo, Brazil

*Corresponding Author**:** Adriana Franco Paes Leme,

Laboratório Nacional de Biociências, LNBio, Centro Nacional de Pesquisa em Energia e Materiais – Rua Giuseppe Maximo Scolfaro, 10000, Polo II de Alta Tecnologia, CEP 13083-970, Campinas, São Paulo, Brazil.

Phone: +55 19 3512-1118; Fax: +55 19 3512-1006; e-mail: adriana.paesleme@lnbio.cnpem.br

Summary

Page 2 Fig S1_Survival analysis of transcript levels from predicted peptidases using PROGgene tool

Page 3 Fig S2. Correlation between transcript levels of predicted peptidases and prognostic features on TCGA clinical data

Page 12 Fig S3_Correlation of saliva proteins with lymph node metastasis status

Page 13 Fig S4_SRM verification of AHSG protein levels in saliva via HTLNQIDEVK peptide

Page 14 **Fig S5_Response of SPINK5 peptides during SRM assay development**

Legend to Figures

Fig S1. Survival analysis on the transcript levels of tissue peptidases using PROGgene tool. Survival curves correlating patterns of protease expression in head and neck tumour tissues and patient prognostic. High (–) expression of *CAPN1*, *CAPN2*, *CTSB* and *MMP11* correlated with reduced overall survival (<40% in 5 years, **A-D**), whilst low (–) levels of *MMP25* transcripts lowers 5-years overall survival (**E-F**). Higher *CAPN1* expression also correlates with reduced metastasis free survival (**G**) whereas relapse free survival is diminished when *MMP25* and *TMPRSS6* expression are low (**H-I**).

Fig S2. Correlation between transcript levels of predicted peptidases and prognostic features on TCGA clinical data. For each peptidase correlated with prognostic features histograms are presented to illustrate the distribution of transcript expression values (log2) within the groups. Normal distribution of the data was tested using Shapiro-Wilk α=0.05). Two-group comparison on parametric data were performed Welch's test whereas non-parametric data were analysed with Kruskal-Wallis. Significance threshold p-value ≤ 0.05

Fig S3. Correlation of saliva proteins with lymph node metastasis status. Three differentially abundant proteins with reduced levels in pN+ saliva were correlated with lymph node metastasis. Significance defined as ANOVA p-value ≤ 0.05.

Fig S4. SRM quantification of AHSG via HTLNQIDEVK peptide. Saliva levels of fetuin A (AHSG) inferred via HTLNQIDEVK peptide across an independent forty-patient cohort. No group differences were observed when **(A)** Nested ANOVA (p-value = 0.7534) or (**B**) Mann-Whitney (p-value=0.0517) group comparisons were performed, as result of the lack of consistent signal in the light channel indicating that the peptide was below the limit of detection.

Fig S5. Extracted ion chromatograms of SPINK5 peptides during SRM method development. FFQSLDGIMFINK(+8Da) peptide confidently detected when 2.5 pmol was loaded in the system in the absence of background matrix **(A)**. In this case, a scheduled acquisition of 1 µg saliva digest spiked with 5 pmol of SIL FFQSLDGIMFINK(+8Da) provided consistent signal in the light and heavy channel **(B)**. Conversely, ATAPTELNCDDFK(+8Da) **(C)** and EAVQELCSEYR(+10Da) **(D)** peptides were not confidently detected even when large amounts (about 5 pmol of crude peptide) of stable isotope labeled synthetic peptides were loaded in the system, in the absence of background matrix. For this reason, only the peptide FFQSLDGIMFINK was monitored in the scheduled SRM assay of the 40 OSCC patient's cohort.

Fig S1_Survival analysis of transcript levels from predicted peptidases using PROGgene tool

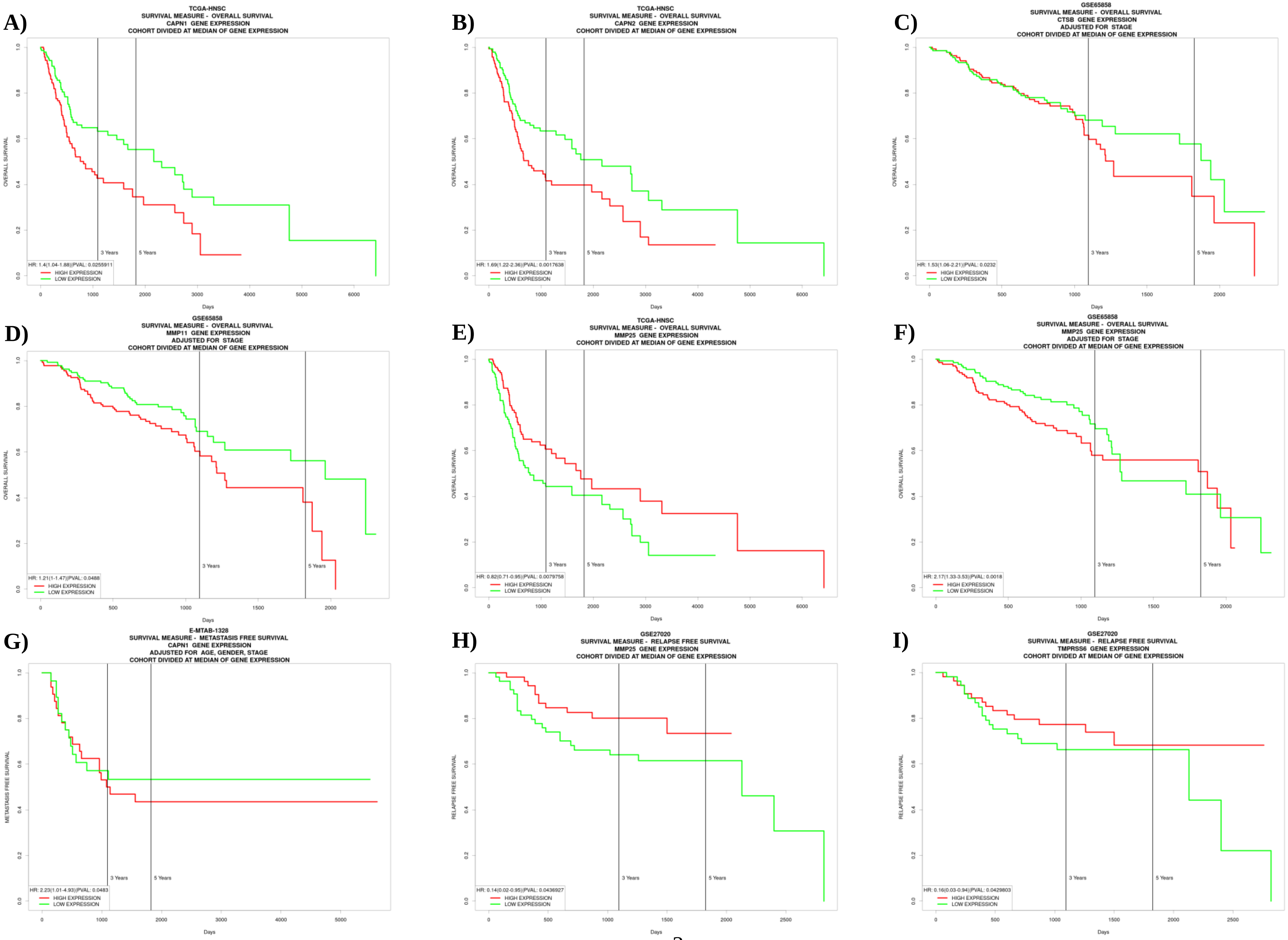

Fig S2. Correlation between transcript levels of predicted peptidases and prognostic features on TCGA clinical data

Cathepsin D – CTSD *vs* vital status (n=120)

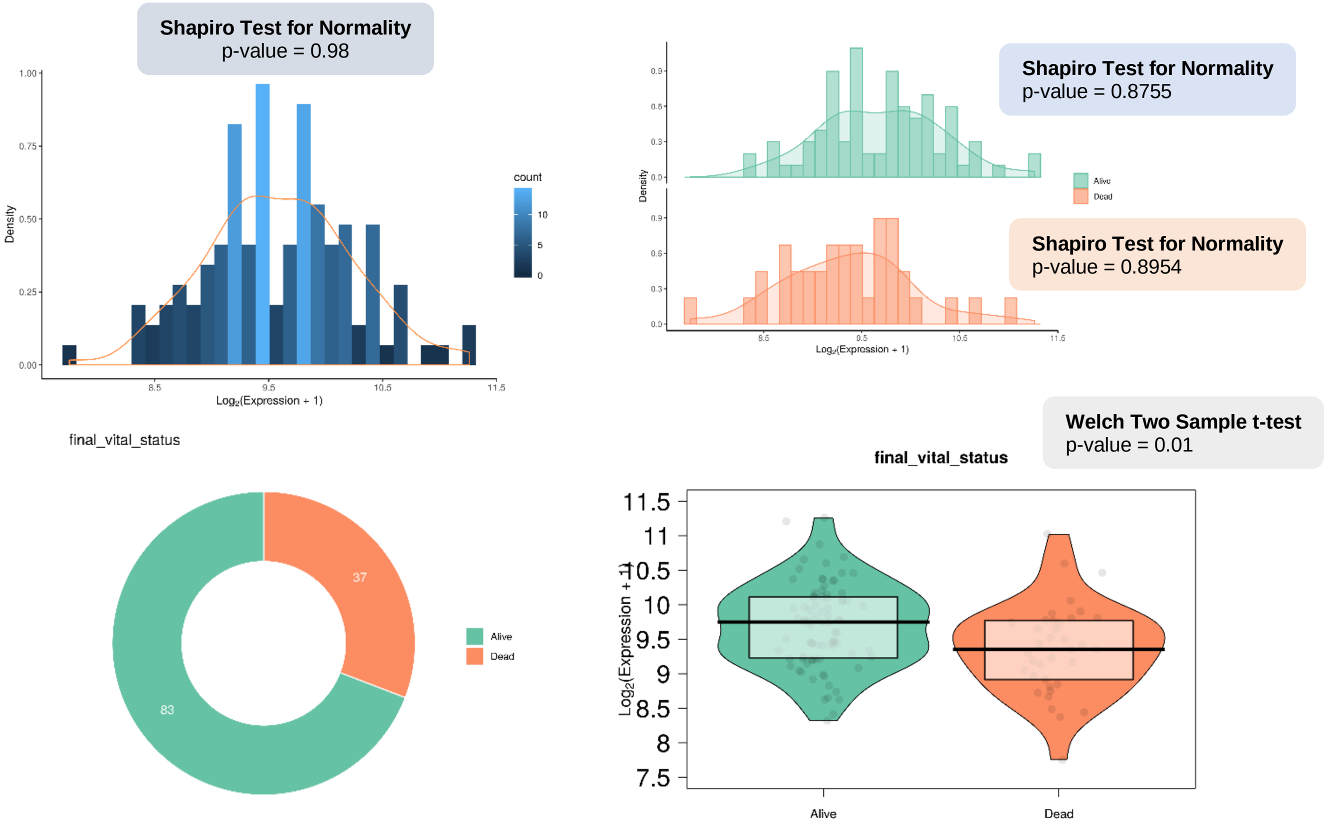

Cathepsin D – CTSD *vs* tumour recurrence (n=104)

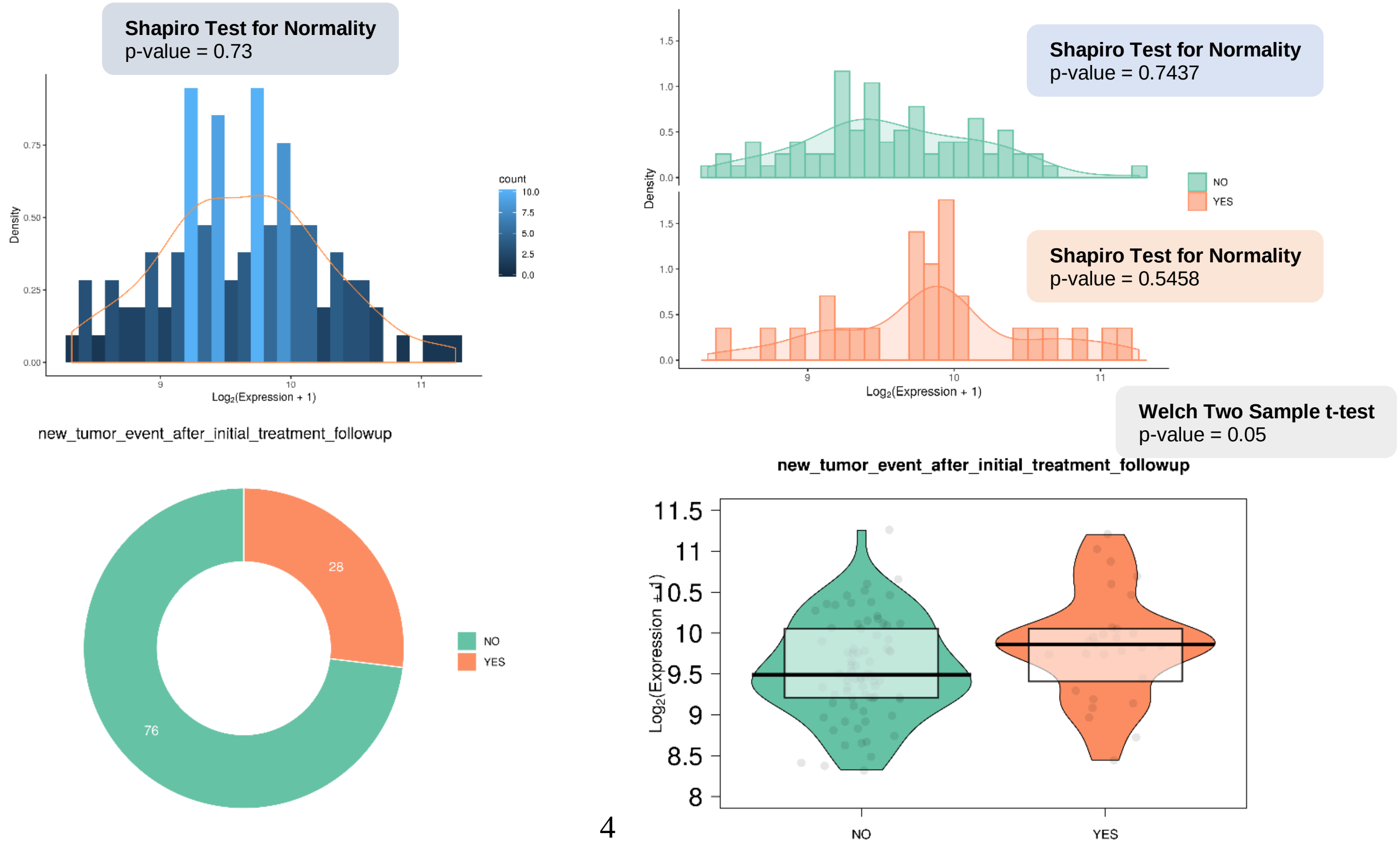

Cathepsin S – CTSS *vs* tumour recurrence (n=104)

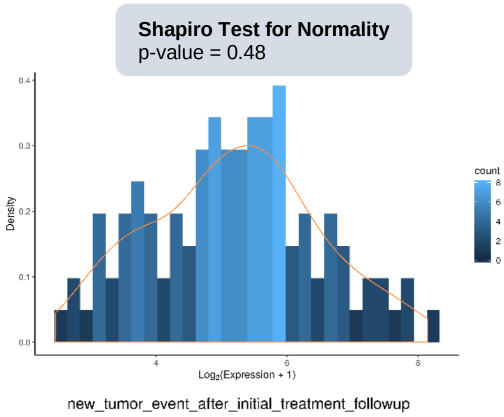

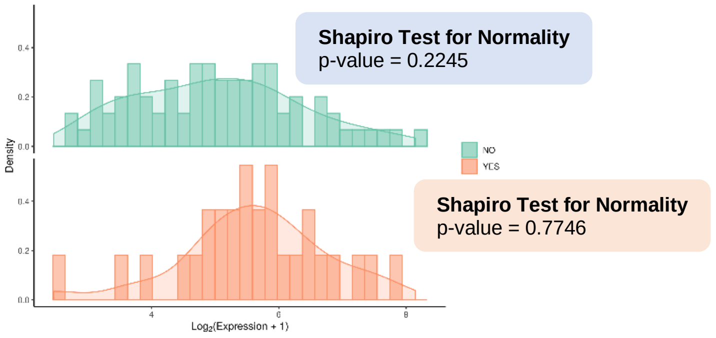

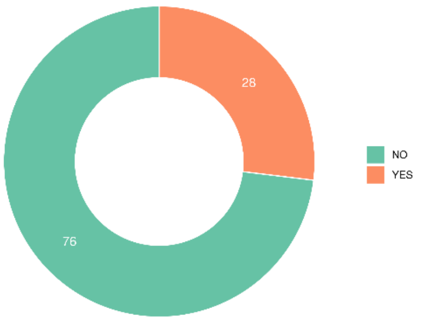

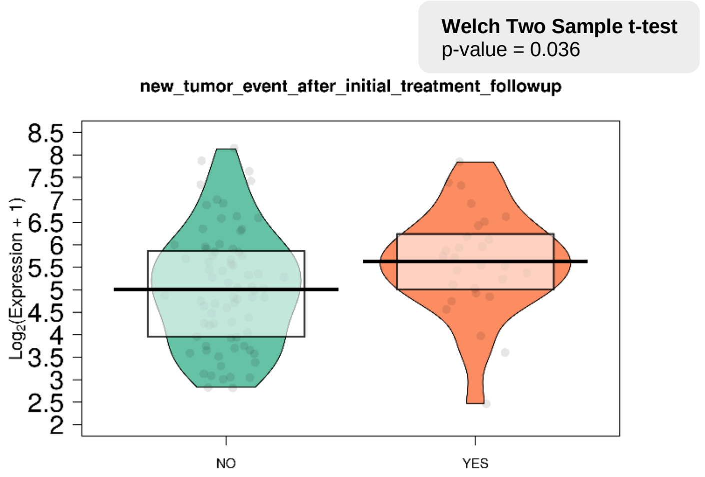

Cathepsin S – CTSS *vs* perineural invasion (n=92)

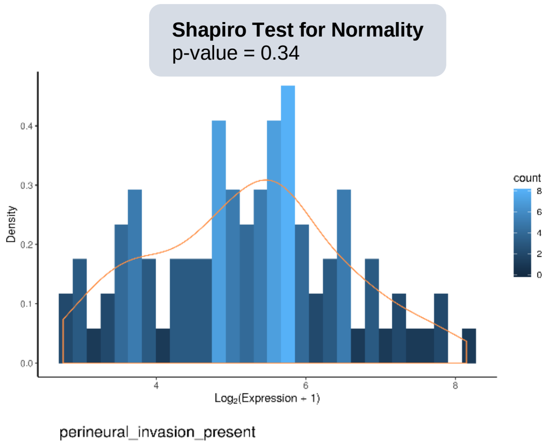

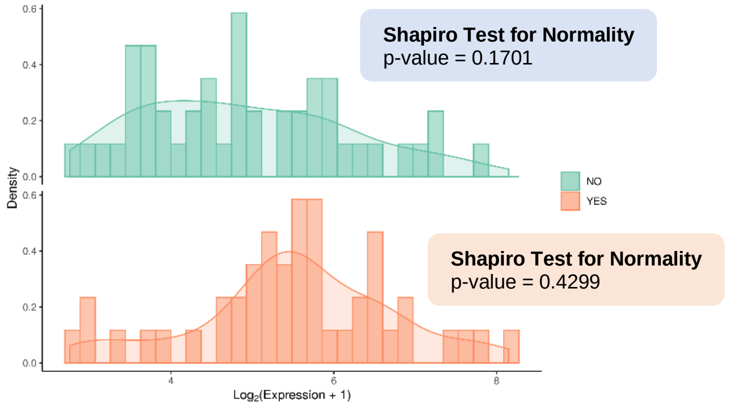

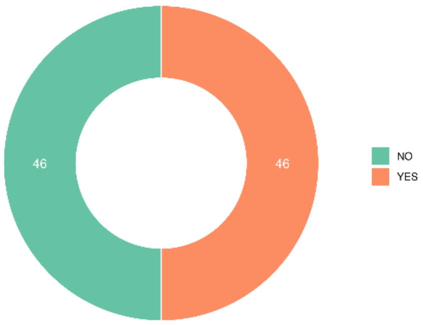

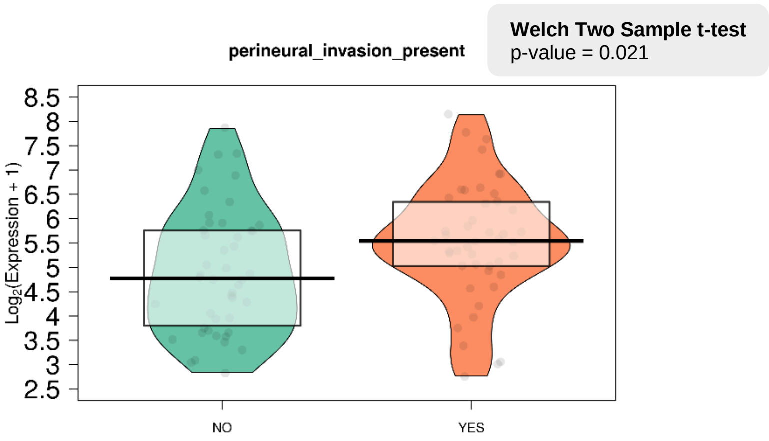

Calpain 1 - CAPN1 *vs* tumour recurrence (n=104)

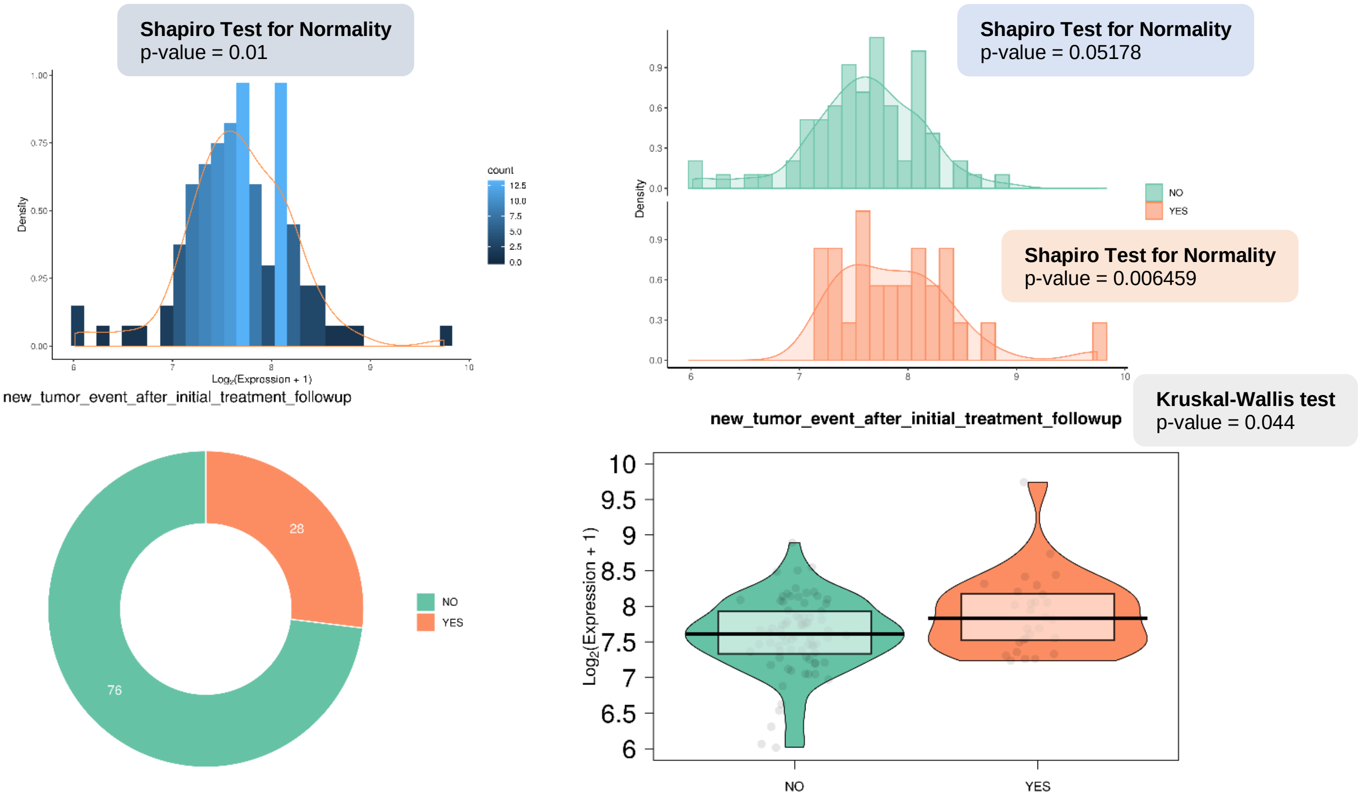

Calpain 2 - CAPN2 *vs* tumour recurrence (n=104)

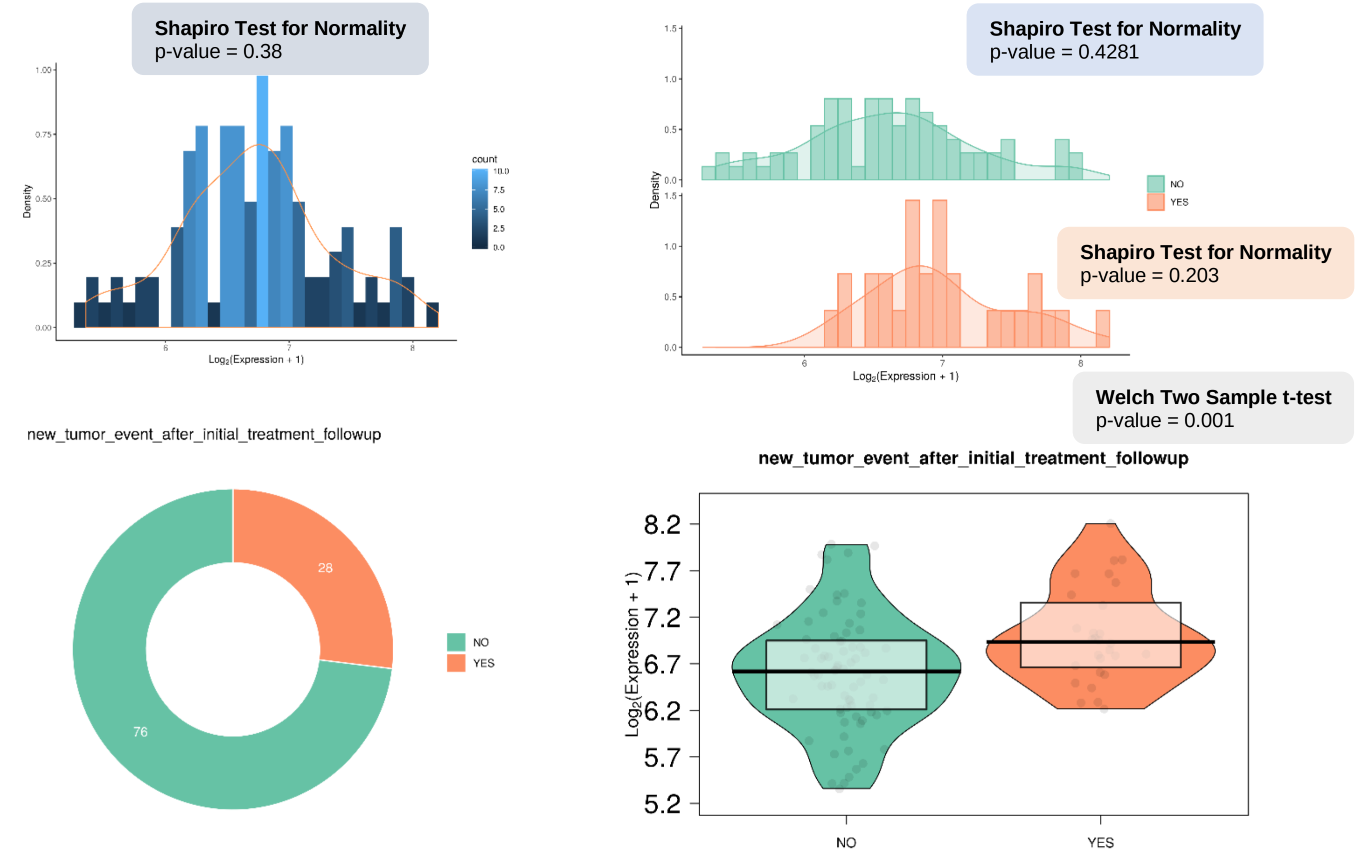

Calpain 2 - CAPN2 *vs* perineural invasion (n=92)

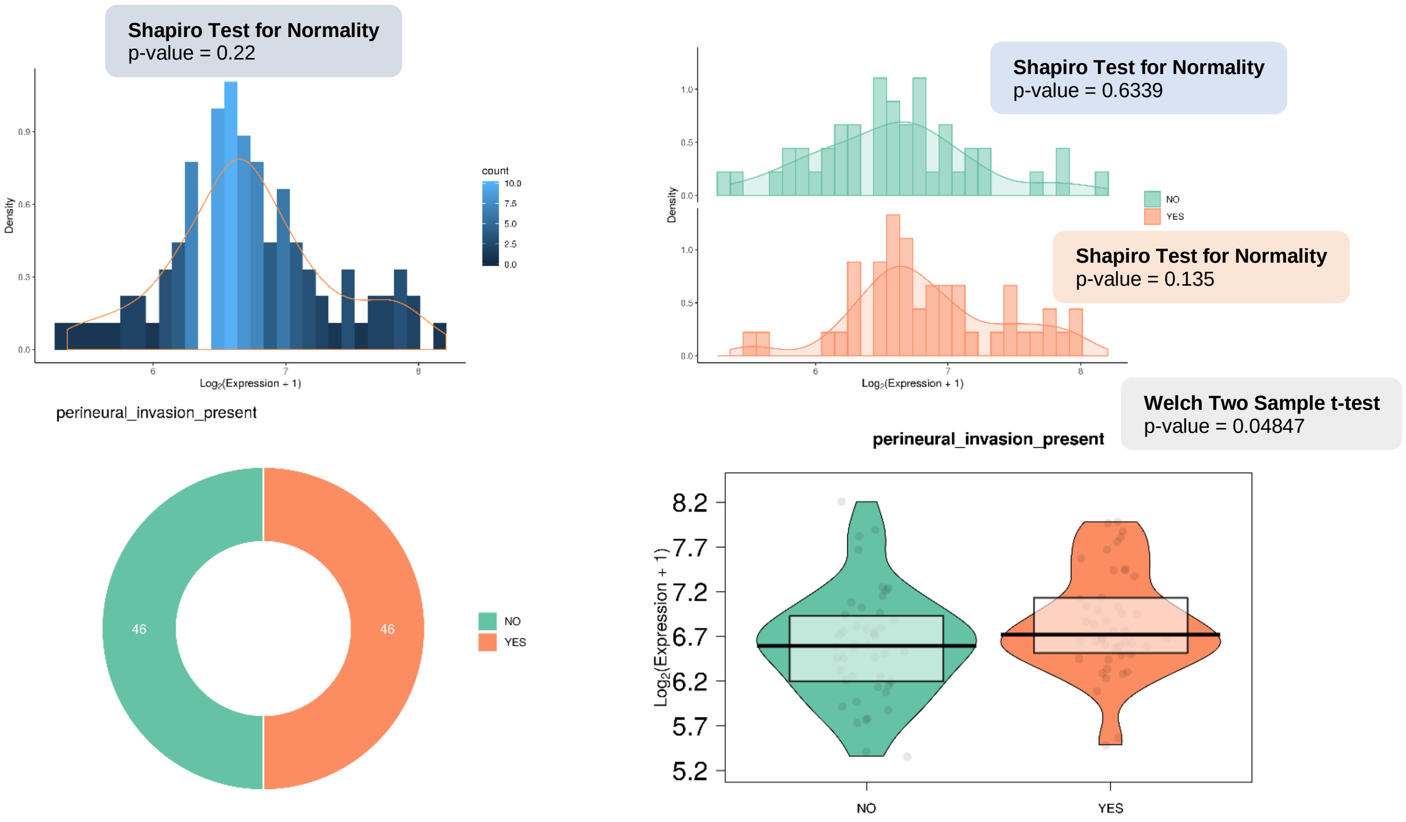

Calpain 2 - CAPN2 *vs* extracapsular extension (n=96)

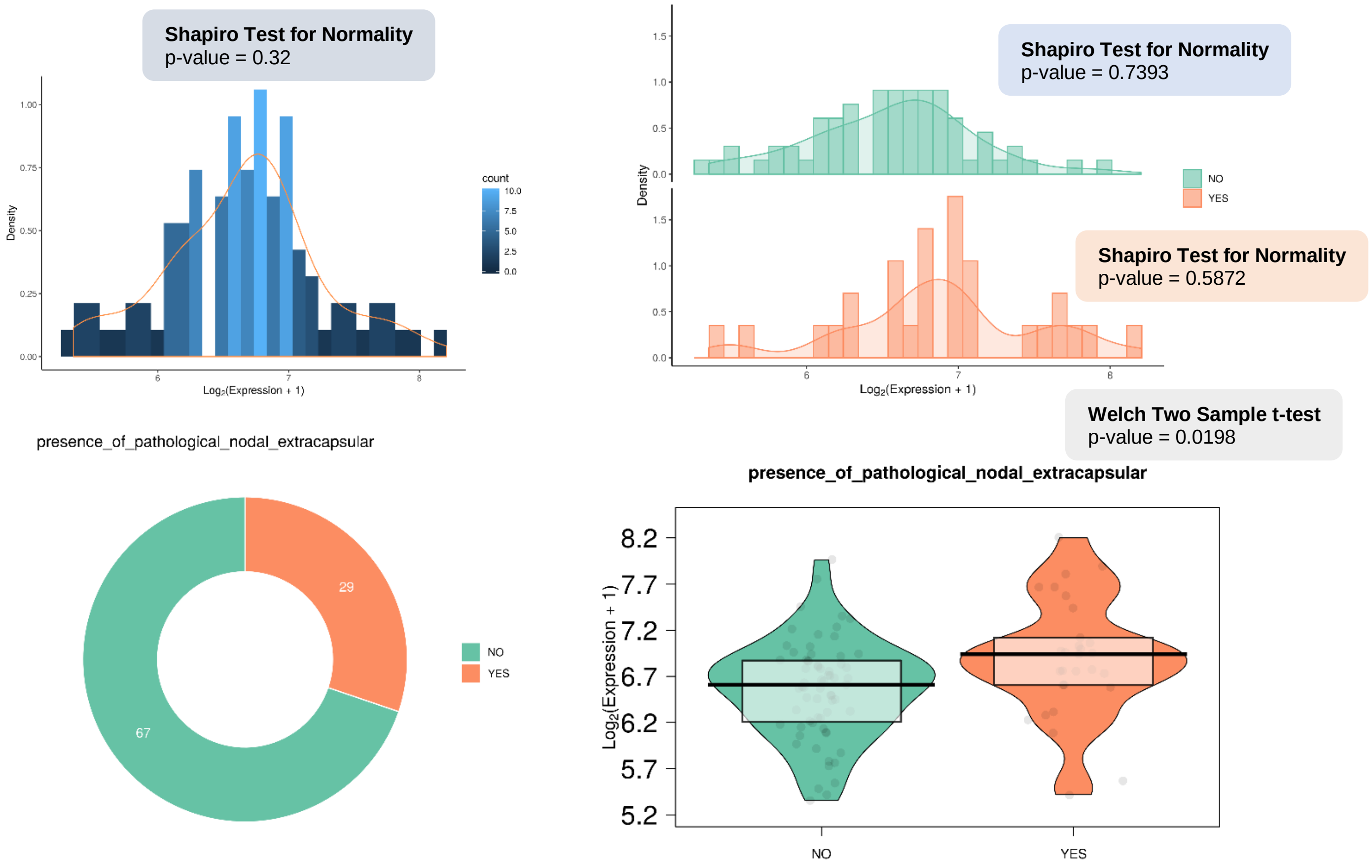

Meprin A - MEP1A *vs* tumour recurrence (n=104)

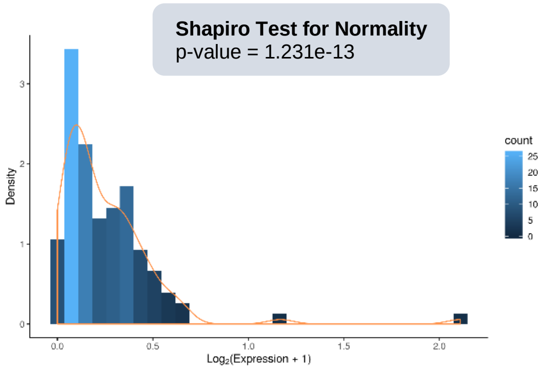

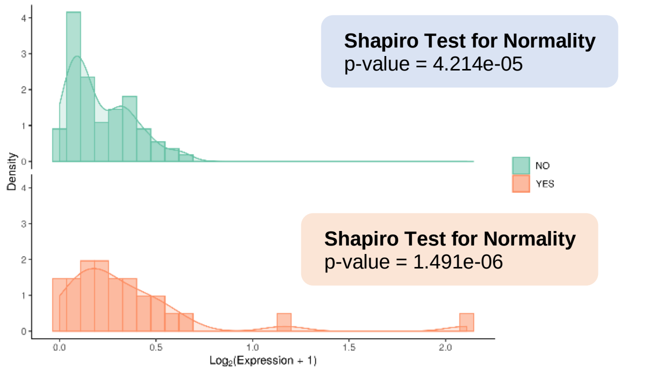

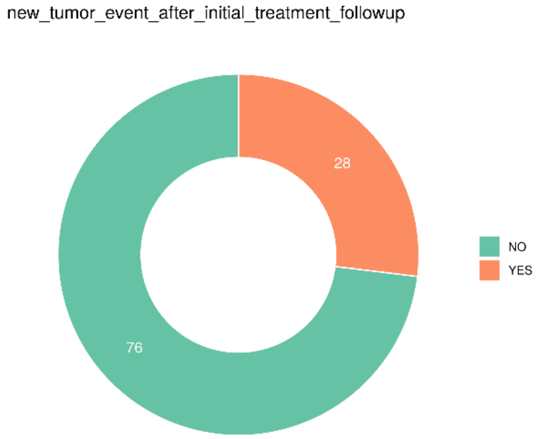

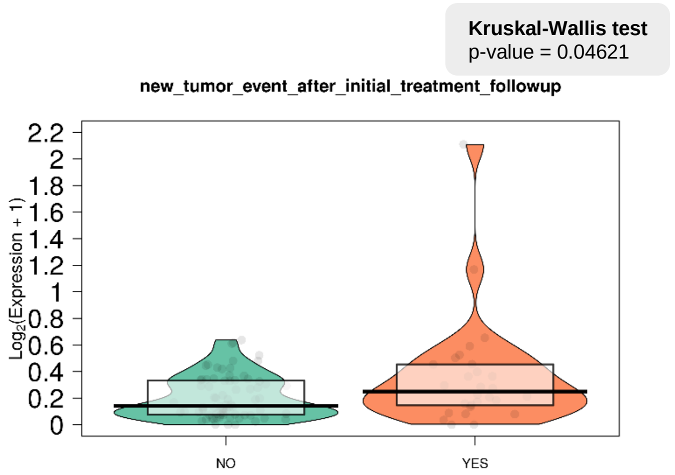

Meprin A - MEP1A *vs* pathologic T grade (n=120)

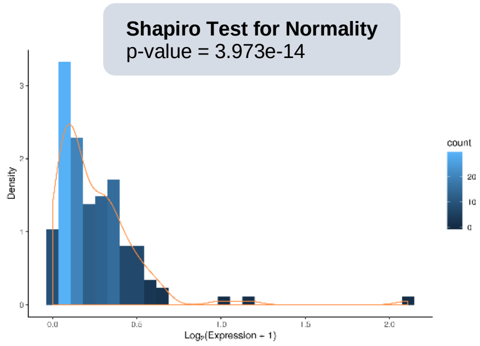

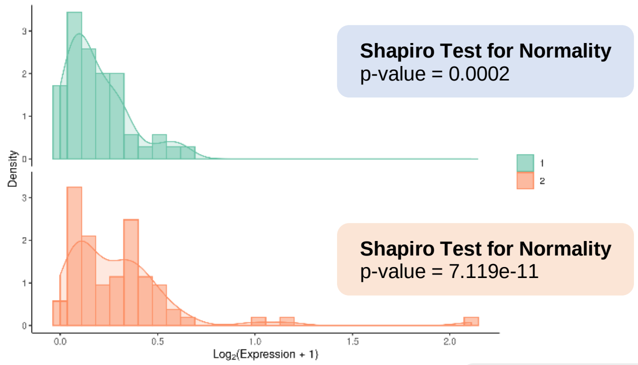

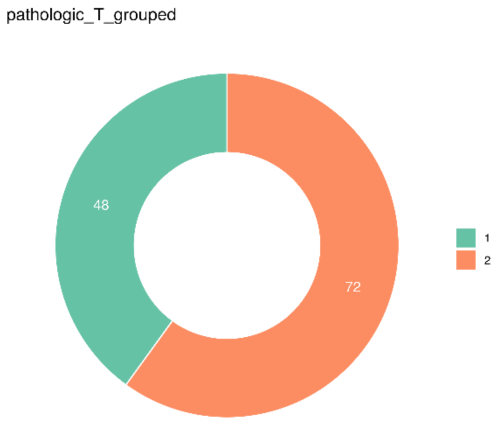

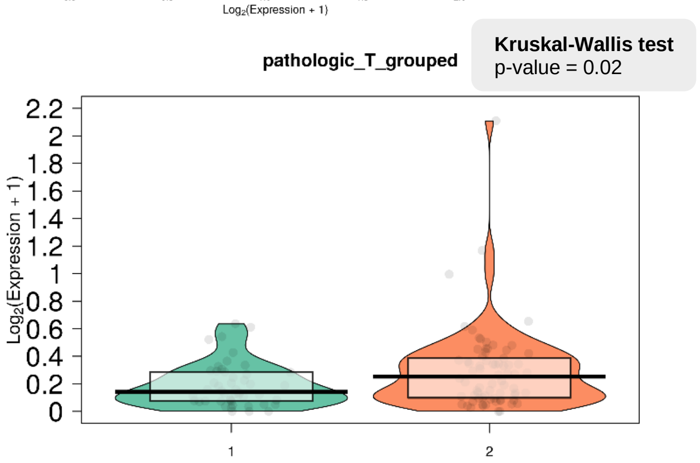

Matrix metallopeptidase 11 - MMP11 *vs* pathologic N status (n=118)

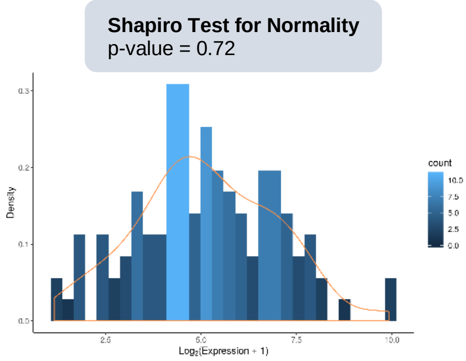

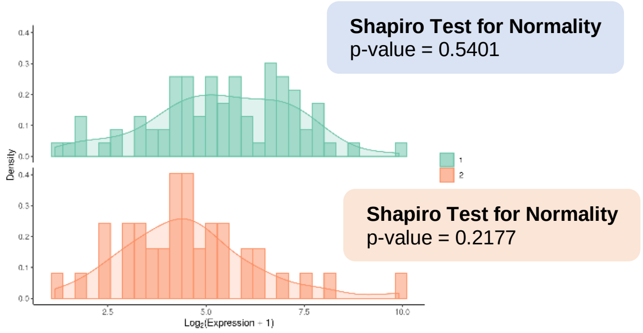

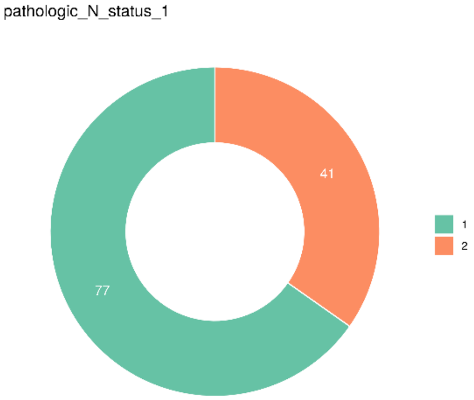

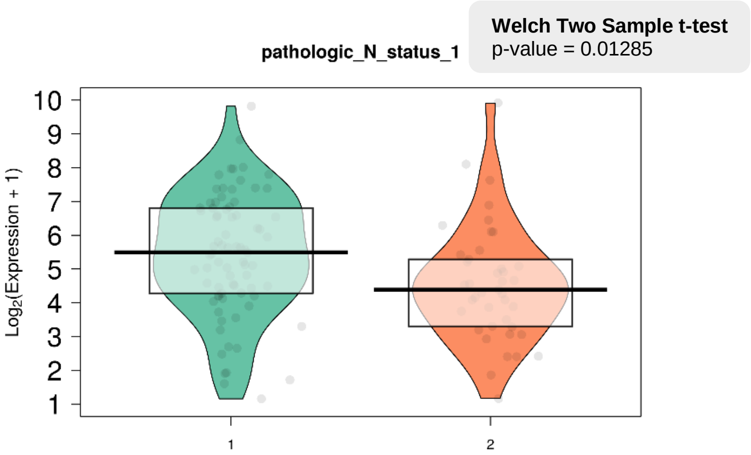

Matrix metallopeptidase 11 - MMP11 *vs* pathologic T grade (n=120)

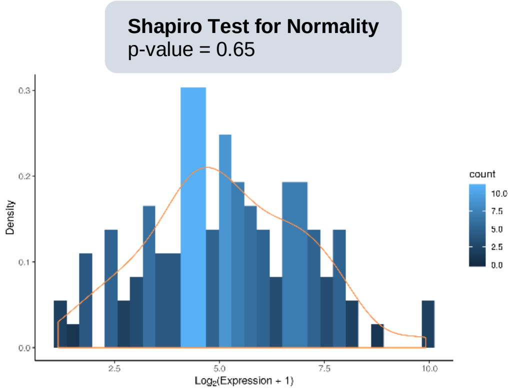

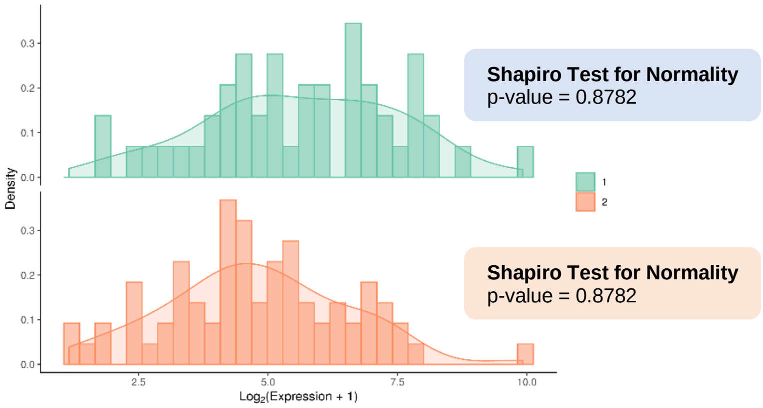

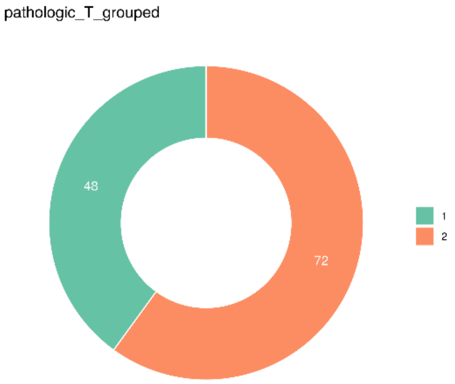

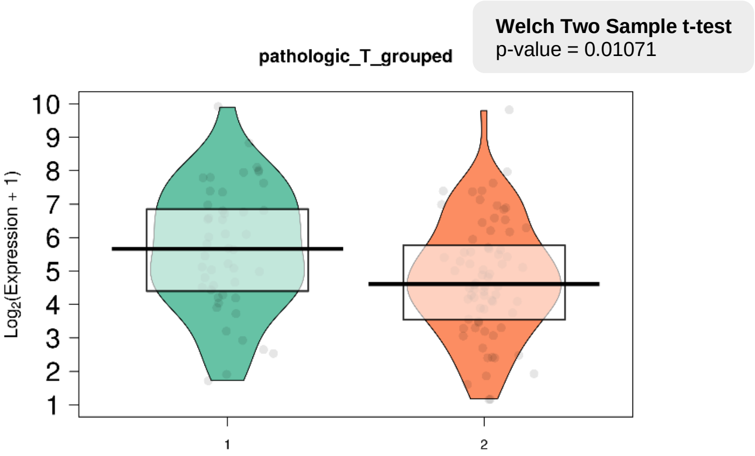

Cathepsin E – CTSE *vs* pathologic T grade (n=120)

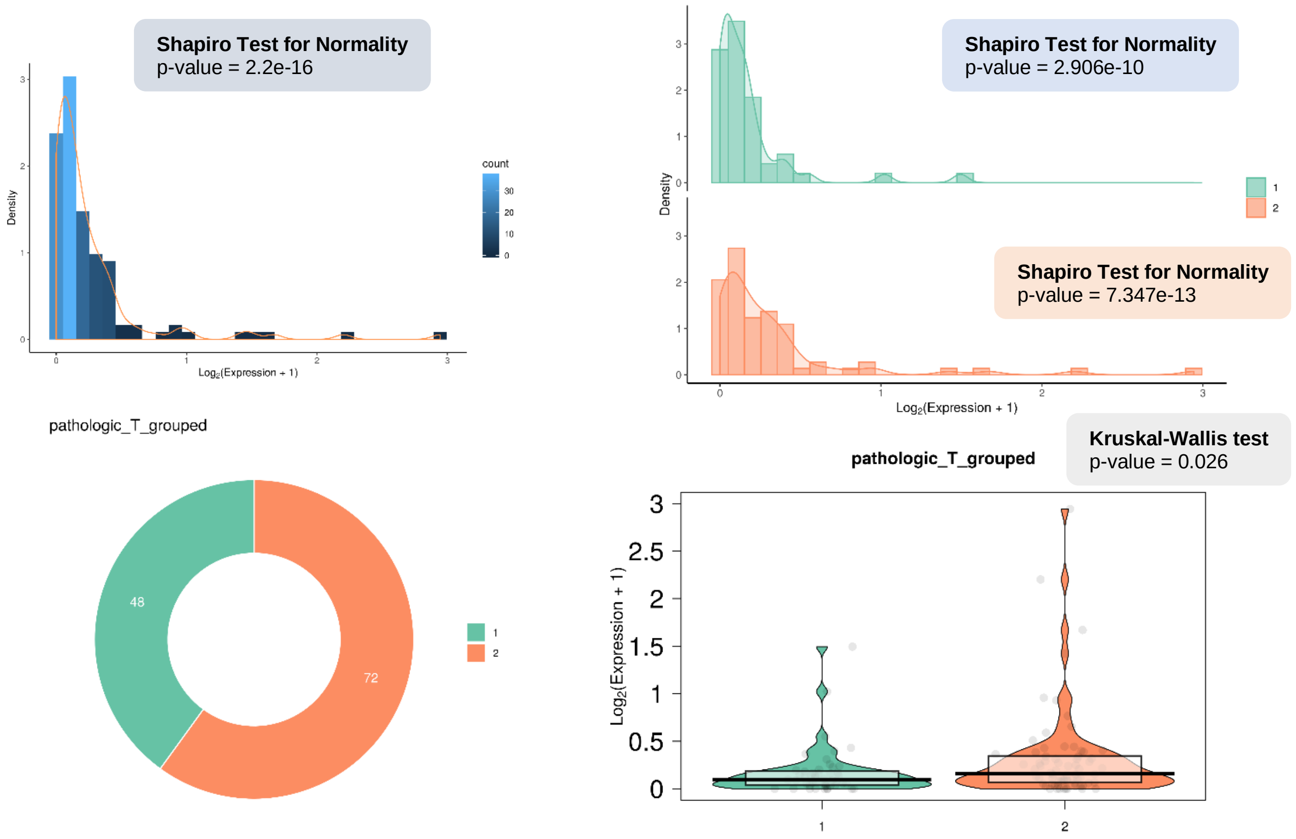

Cathepsin K – CTSK *vs* pathologic T grade (n=120)

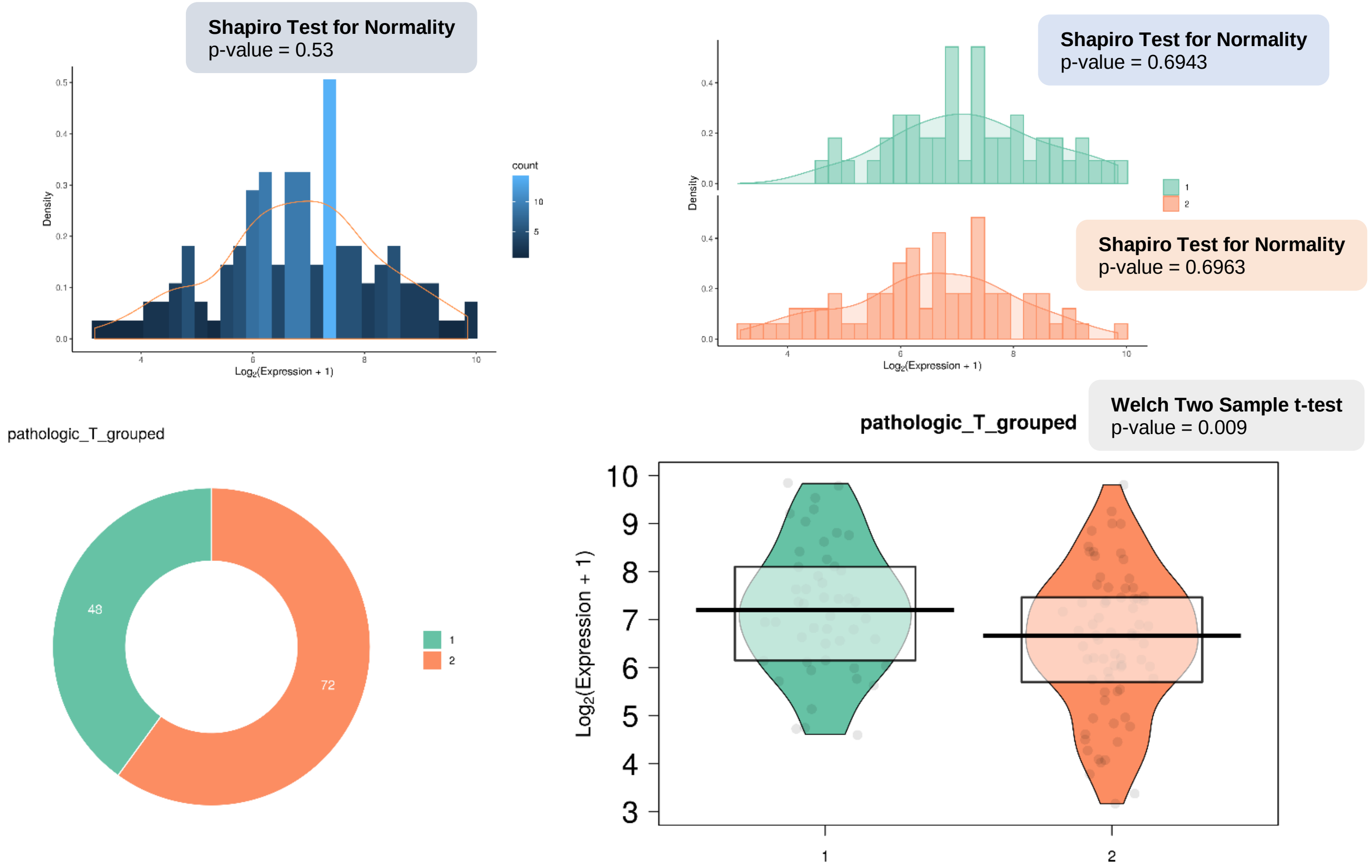

Matrix metalloproteinase 2 - MMP2 *vs* pathologic T grade (n=120)

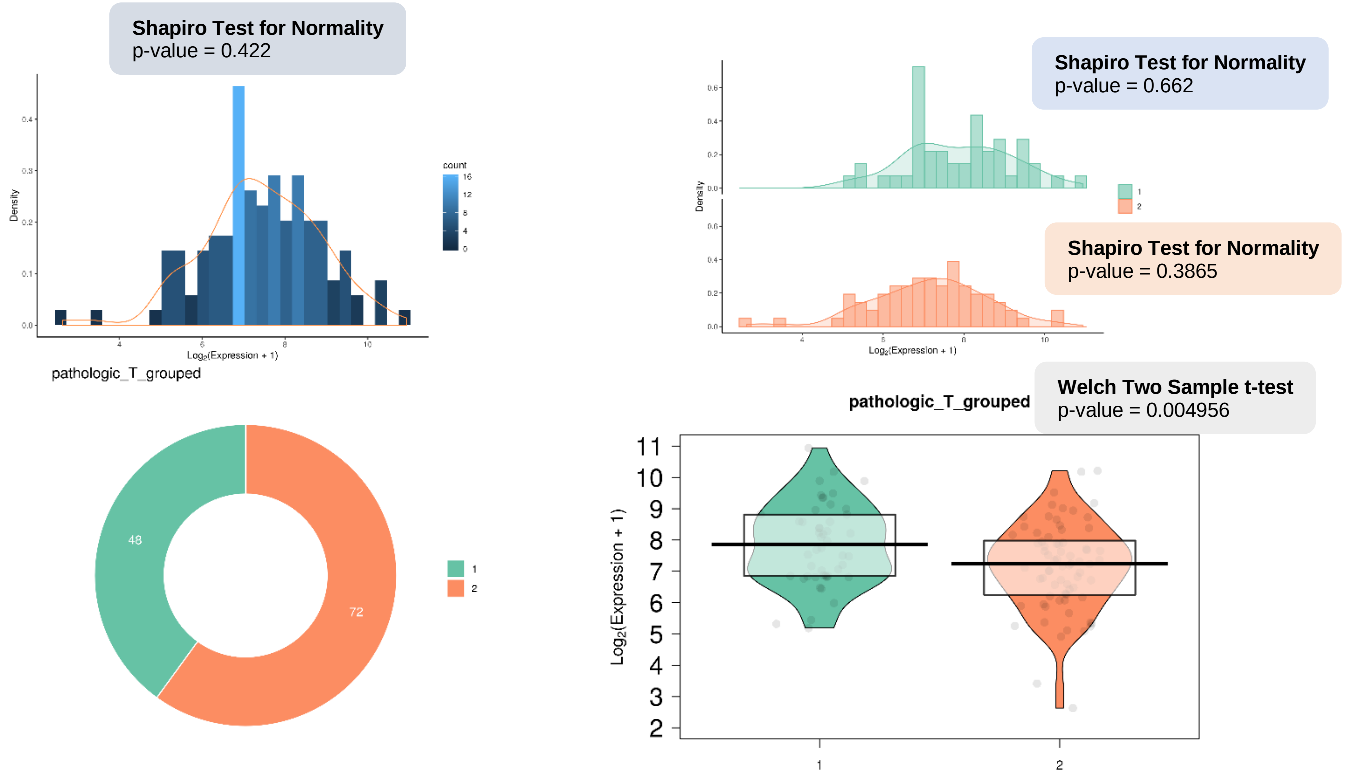

Matrix metalloproteinase 25 - MMP25 *vs* perineural invasion (n=92)

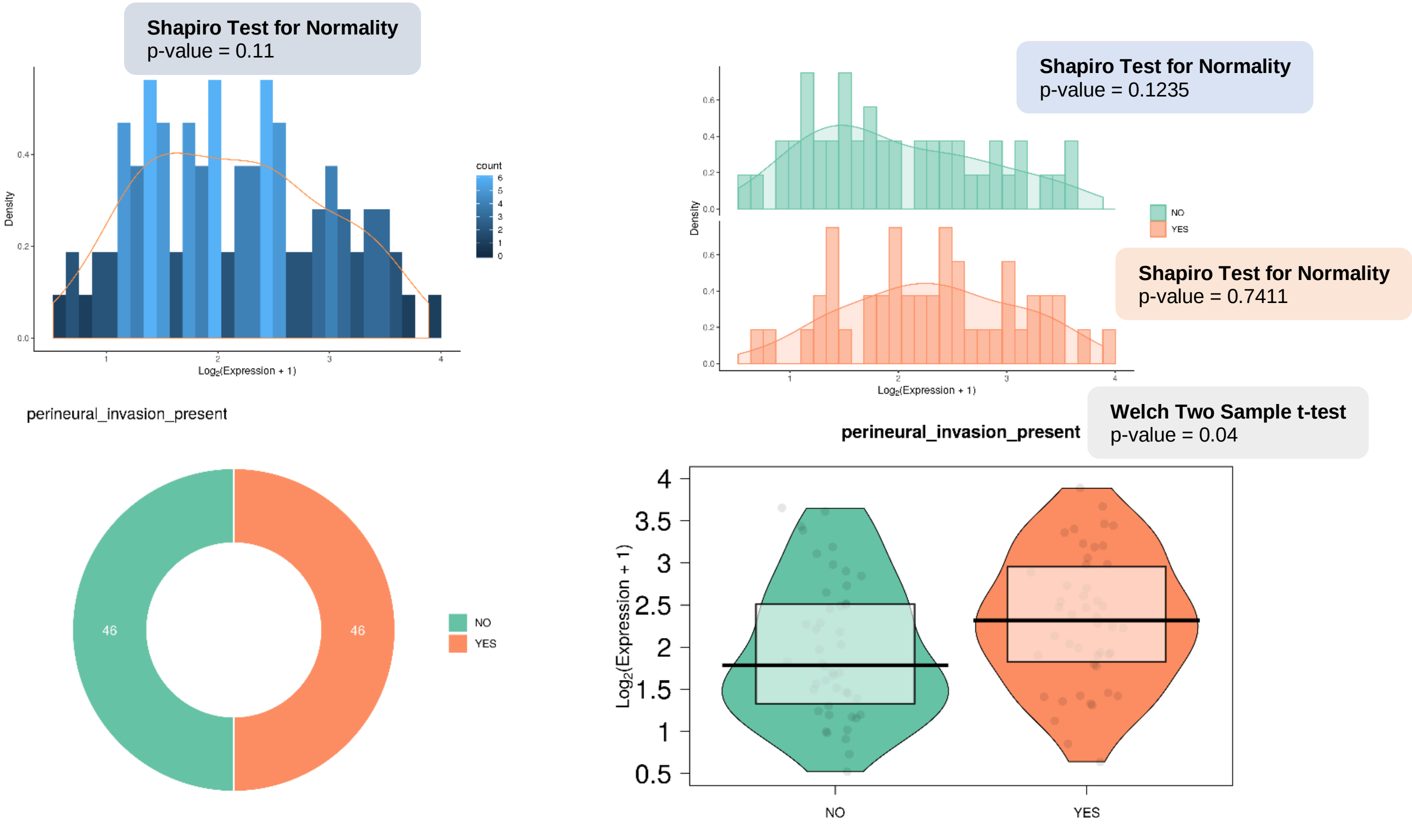

Fig S3_Correlation of saliva proteins with lymph node metastasis status

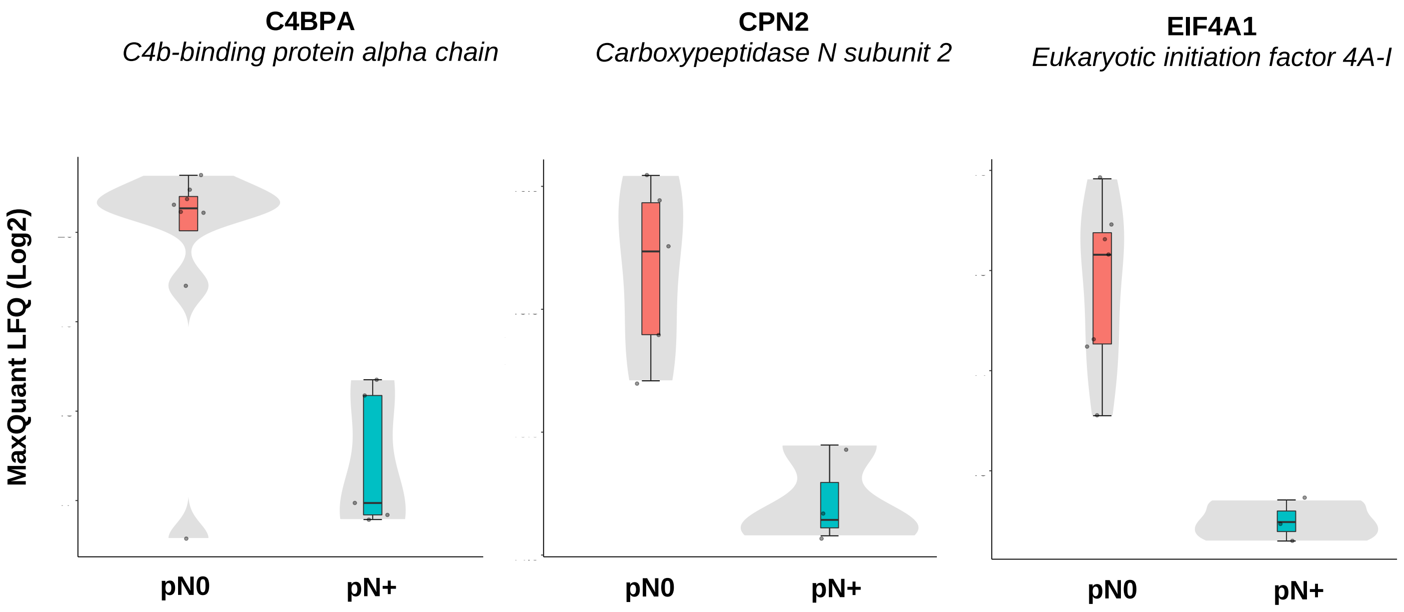

Fig S4_SRM verification of AHSG protein levels in saliva via HTLNQIDEVK peptide

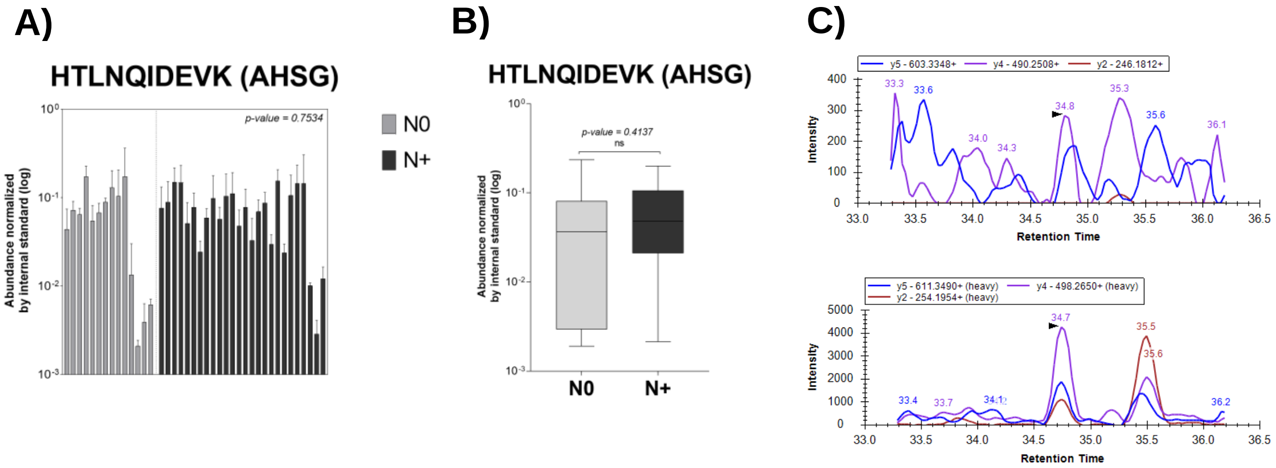

Fig S5_Response of SPINK5 peptides during SRM assay development

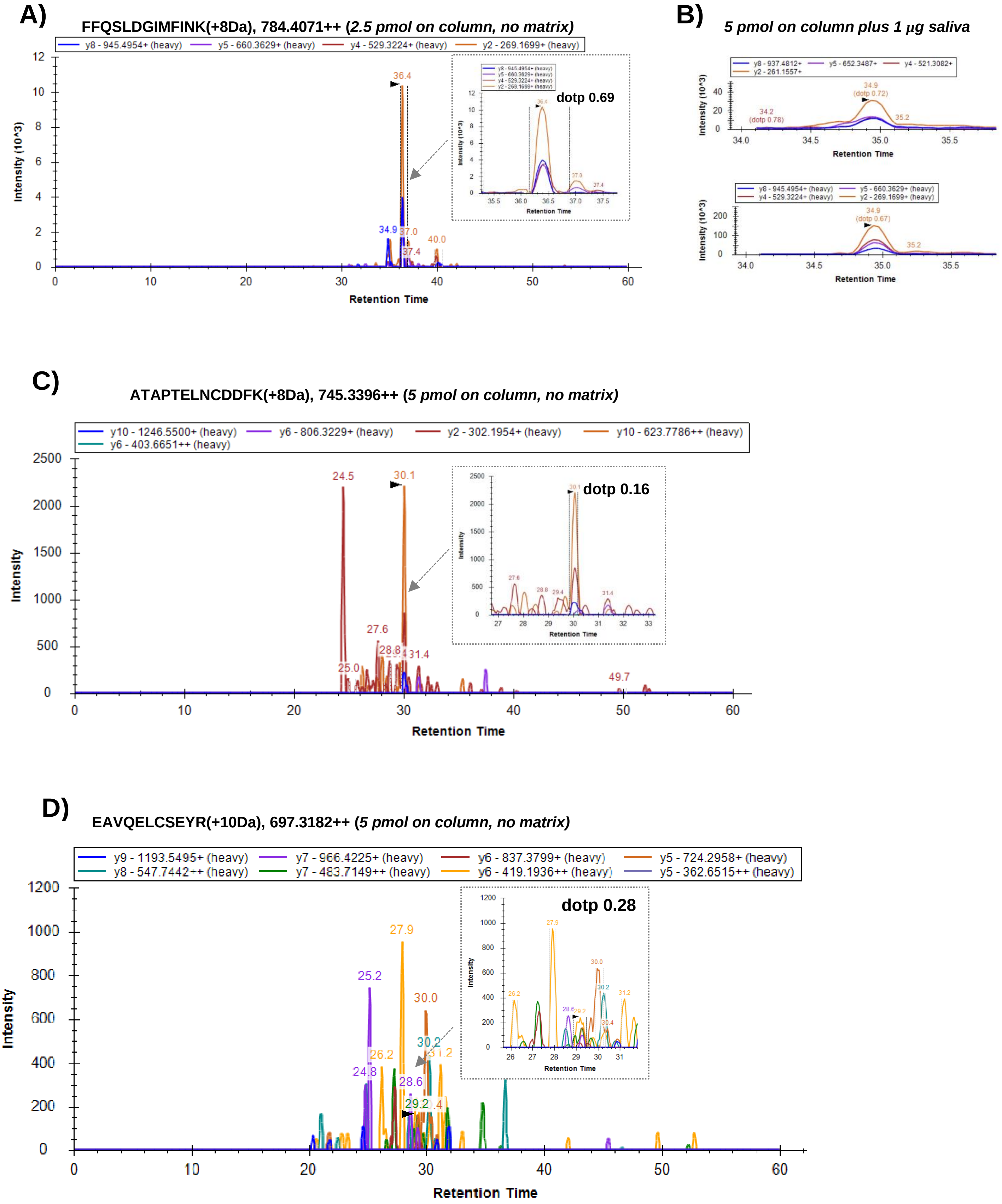

Supplement: Supplemental Figures S1 to S5 [file mmc2.pdf]
